# Five-variable nomogram including PR interval and left atrial appendage flow velocity predicts atrial fibrillation recurrence after cryoballoon ablation

**DOI:** 10.1038/s41598-026-35653-9

**Published:** 2026-01-17

**Authors:** Qiqiang Jie, Weichun Qian, Haibo Jia, Fengfu Zhang, Jianping Wang

**Affiliations:** 1 Department of Cardiology, Nanjing First Hospital, Nanjing Medical University, Nanjing, China; 2 Department of Geriatrics, Nanjing First Hospital, Nanjing Medical University, Nanjing, China

**Keywords:** Atrial fibrillation, Cryoballoon ablation, Predictive nomogram, PR interval, Left atrial appendage flow velocity, Risk stratification, Interventional cardiology, Predictive markers, Atrial fibrillation

## Abstract

Cryoballoon ablation for atrial fibrillation (AF) carries a non-negligible 1-year recurrence risk, and existing risk models often omit electrocardiographic and echocardiographic features. We aimed to develop and internally validate an AF recurrence prediction model that integrates the PR interval and echocardiographic measures—left atrial dimension (LAD) and left atrial appendage flow velocity (LAAFV)—with clinical characteristics. In this single-center retrospective cohort of 757 patients who underwent first-time cryoballoon ablation (2017–2023), participants were randomly divided into training (70%) and validation (30%) sets. Candidate predictors were selected via least absolute shrinkage and selection operator (LASSO) and random forest. Model performance was evaluated at prespecified 12- and 24-month horizons with time-dependent receiver operating characteristic (ROC) curves, calibration, decision curve analysis, and Kaplan–Meier estimates. Fiveindependently associated predictors were identified: female sex, persistent AF, prolonged PR interval, increased LAD, and reduced LAAFV. A multivariable Cox proportional hazards model was used to construct the nomogram. Head-to-head benchmarking against APPLE, SUCCESS, PAT2C2H, HATCH, BASE-AF2, and CHA₂DS₂-VASc was performed within the same participants via two-sided paired DeLong tests with Holm correction. The nomogram showed strong discrimination (AUC 0.81 and 0.83 in training; 0.82 and 0.80 in validation at 12 and 24 months, respectively) and outperformed all comparators at both horizons (Holm-adjusted P < 0.01). Risk stratification separated the low-, intermediate-, and high-risk groups, with 24-month recurrence-free survival rates of approximately 80%, 45%, and 20%, respectively. By combining structural and functional atrial parameters with clinical features, this tool enhances the prediction of AF recurrence after cryoballoon ablation and may inform individualized postablation management. Prospective multicenter external validation is warranted.

**Supplementary Information:**

The online version contains supplementary material available at 10.1038/s41598-026-35653-9.

## Introduction

Atrial fibrillation (AF) is one of the most common heart rhythm disorders worldwide and is associated with a fivefold greater risk of stroke and a markedly increased risk of heart failure^1,2^. Recent Global Burden of Disease (GBD) 2019–based analyses indicate that the number of incident casesof AF/AFL increased by ~103.9% and the number of prevalent cases increased by ~111.0% from 1990 to 2019^3^. Despite advances in therapeutic approaches, including radiofrequency catheter ablation (RFCA) and cryoballoon ablation, AF recurrence remains a significant clinical challenge, with reported recurrence rates of approximately 30–50% within the first year after ablation^2,3^. This high recurrence rate highlights the urgent need for accurate predictive tools to optimize postablation management and improve patient outcomes.

The likelihood of AF recurrence is influenced by a variety of interconnected elements, including the remodeling of the left atrium’s structure and electrical properties; comorbidities such as hypertension, diabetes, and chronic obstructive pulmonary disease; and demographic factors^4–8^. Moreover, specific laboratory markers, including elevated brain natriuretic peptide (BNP), serum creatinine, and cardiac troponins, have demonstrated significant associations with recurrence risk^4^. This complex interplay of factors underscores the necessity for comprehensive risk assessment strategies.

Models such as CHADS_2_ and CHA_2_DS_2_-VASc are beneficial for overall risk stratification but show limited effectiveness in predicting recurrence following ablation^2^. Several ablation-specific risk scores—such as APPLE^9^, SUCCESS, PAT2C2H^10^, HATCH, and BASE-AF2^11^—have been developed to estimate recurrence risk after AF ablation, including in clinical practice following cryoballoon ablation^12^. However, these tools rely largely on clinical and structural variables and rarely incorporate electrocardiographic or functional echocardiographic markers, such as the PR interval and left atrial appendage flow velocity (LAAFV), which reflect atrial conduction and mechanical function. New findings indicate that prolonged PR intervals, reflecting delayed atrial conduction, are associated with an approximately 30% higher risk of AF development and/or recurrence^13,14^. Furthermore, the left atrial appendage flow velocity (LAAFV) has emerged as a vital echocardiographic marker for both thrombus formation risk and AF recurrence prediction^15^.

To address these limitations, we developed a clinical prediction model that integrates conventional clinical features (female sex and persistent AF) with electrocardiographic (PR interval) and echocardiographic markers—left atrial dimension (LAD) and LAAFV. These parameters were selected on the basis of their established associations with AF recurrence and ability to reflect both structural and functional atrial remodeling. This integrated approach aimed to increase the accuracy of recurrence risk stratification following cryoballoon ablation. The clinical usefulness of this model is its ability to identify high-risk patients and tailor postprocedural management plans such as improved follow-up schedules and prompt interventions. This detailed risk-evaluation strategy allows clinicians to better adapt therapeutic approaches, potentially leading to improved long-term outcomes in patients undergoing AF ablation. To contextualize performance within the cryoballoon ablation (CBA) cohort, we prespecified head-to-head benchmarking at 12 and 24 months against APPLE, SUCCESS, PAT2C2H, HATCH, BASE-AF2, and CHA₂DS₂-VASc using two-sided, paired DeLong tests with Holm adjustment (same participants per horizon).

## Results

### Baseline characteristics

**Fig. 1:**
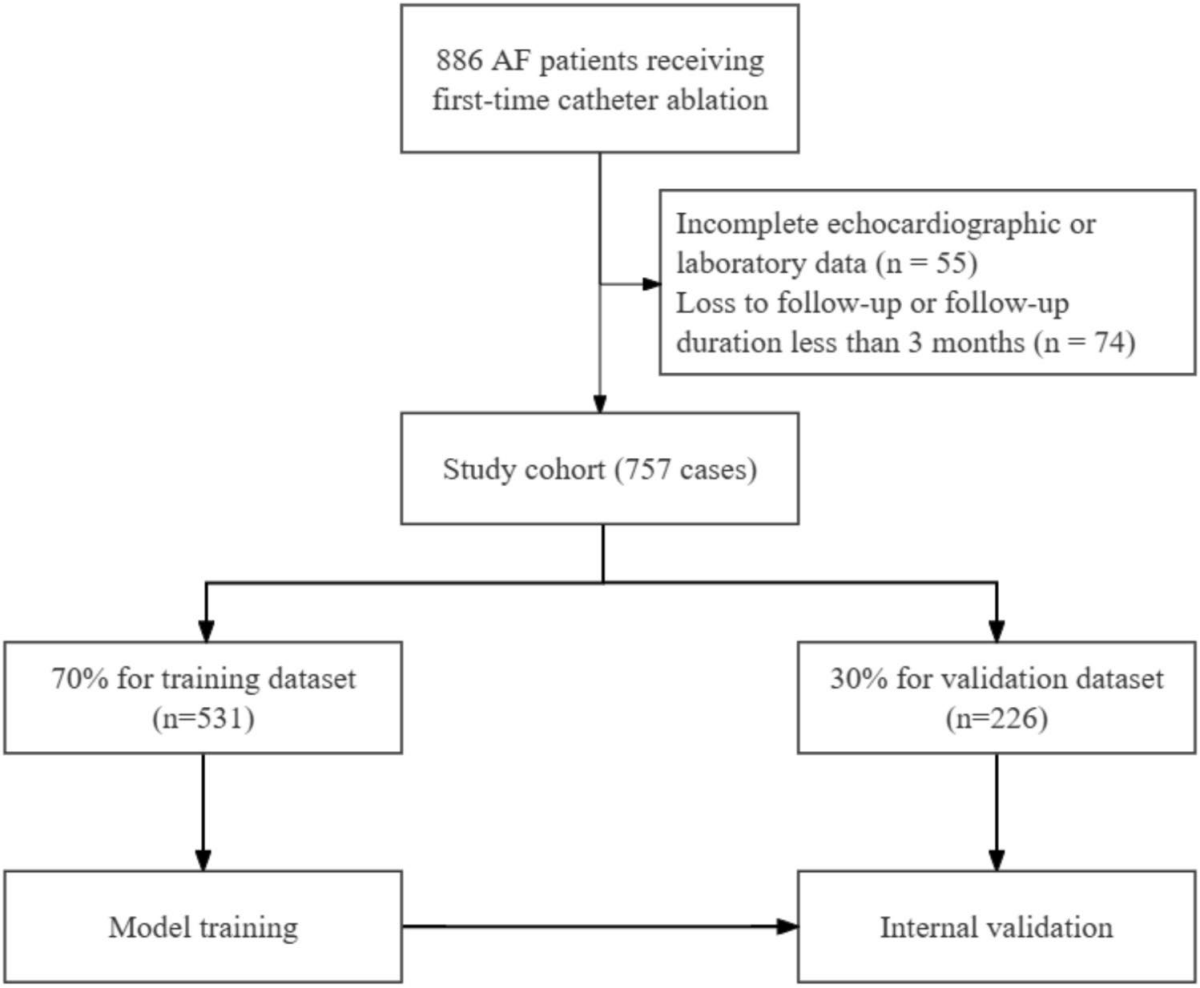
Flowchart of patient selection and random data partitioning. A total of 886 atrial fibrillation (AF) patients who underwent first-time cryoballoon ablation were assessed for eligibility. After 55 patients with incomplete echocardiographic or laboratory data and 74 patients who were lost to follow-up or had a follow-up duration of less than 3 months were excluded, a final study cohort of 757 patients was included. The cohort was randomly allocated into a training dataset (70%, n = 531) for model training and a validation dataset (30%, n = 226) for internal validation.

In this study, 757 patients with AF were enrolled, comprising 309 and 448 patients in the nonrecurrence and recurrence groups, respectively (Figure 1). Demographics and clinical characteristics differed significantly between the groups (Table 1). The proportion of females was greater in the recurrence group (46% vs. 33%, P < 0.001), as was the proportion of persistent AF (44% vs. 30%, P < 0.001). Systolic blood pressure was greater in the recurrence group (133.7 ± 13.9 vs. 130.1 ± 13.0 mmHg, P < 0.001). However, age, BMI, duration of AF, and CHA_2_DS_2_-VASc score were not significantly different between the groups.

**Table 1:** Baseline characteristics of 757 AF patients with and *without* atrial fibrillation recurrence after cryoballoon.

**Characteristic**	**Total** **(n = 757)**	**No Recurrence** **(n = 309)**	**Recurrence** **(n = 448)**	***P* value**
Age (years)	60.2±10.5	61.0±9.9	59.5±10.9	0.10
Female [n (%)]	306 (40)	102 (33)	204 (46)	<0.001
BMI (kg/m^2^)	25.1±3.1	25.2±3.1	25.0±3.1	0.21
persistent AF [n (%)]	239 (32)	67 (22)	172 (38)	<0.001
AF History(months)	25.1±42.7	23.6±43.8	26.1±41.8	0.13
CHA_2_DS_2_-VASc	1.7±1.4	1.7±1.5	1.6±1.4	0.24
SBP (mmHg)	132.2±13.7	130.1±13.0	133.7±13.9	<0.001
DBP (mmHg)	76.4±14.2	76.9±8.8	76.1±16.9	0.02
**Comorbidities [n (%)]**				
Hypertension	390 (52)	161 (52)	229 (51)	0.85
Diabetes	107 (14)	47 (15)	60 (13)	0.55
Stroke/TIA	78 (10)	29 (9)	49 (11)	0.57
CAD	96 (13)	44 (14)	52 (12)	0.34
PCI [n (%)]	31 (4)	14 (5)	17 (4)	0.75
Hyperlipidemia	45 (6)	21 (7)	24 (5)	0.51
Smoking	157 (21)	66 (21)	91 (20)	0.80
Alcohol	121 (16)	43 (14)	78 (17)	0.23
**Laboratory data**				
WBC	5.9±1.5	5.8±1.5	5.9±1.5	0.29
RBC	4.5±0.5	4.5±0.5	4.5±0.5	0.29
Hb(g/L)	136.1±15.6	135.1±15.5	136.8±15.5	0.14
Creatinine (µmol/L)	70.7±15.7	70.6±16.1	70.8±15.5	0.85
Urea (mmol/L)	5.7±1.8	5.7±1.6	5.7±1.9	0.34
Uric acid (µmol/L)	326.9±95.8	328.7±96.7	325.6±95.2	0.63
Total cholesterol (µmol/L)	4.2±1.0	4.2±1.0	4.2±0.9	0.41
Triglyceride (µmol/L)	1.6±0.9	1.6±1.0	1.6±0.9	0.60
HDL-C (µmol/L)	1.1±0.3	1.1±0.3	1.1±0.3	0.81
LDL-C (µmol/L)	2.4±0.8	2.3±0.8	2.4±0.7	0.07
ST2(ng/mL)	4.2±1.0	4.2±1.0	4.2±0.9	0.41
BNP(pg/mL)	387.8±265.9	285.7±210.5	458.2±277.2	<0.001
**Medications [n (%)]**				
Propafenone	175 (23)	69 (22)	106 (24)	0.73
Amiodarone	287 (38)	123 (40)	164 (37)	0.41
Dronedarone	44 (6)	23 (7)	21 (5)	0.15
Wuxin particle	44 (6)	22 (7)	22 (5)	0.26
Beta-blockers	239 (32)	93 (30)	146 (33)	0.52
Rivaroxaban	521 (69)	233 (75)	288 (64)	<0.01
Dabigatran	212 (29)	80 (27)	132 (30)	0.40
Statins	239 (32)	127 (41)	112 (25)	<0.001
ARNIs	44 (6)	25 (8)	19 (4)	0.04
ACEI	50 (7)	23 (7)	27 (6)	0.53
ARBs	190 (25)	84 (27)	106 (24)	0.31
Loop diuretics	13 (2)	7 (2)	6 (1)	0.50
Thiazide diuretics	35 (5)	17 (6)	18 (4)	0.44
Spironolactone	22 (3)	9 (3)	13 (3)	1.00
**Electrocardiograph**				
HR	77.7±10.9	77.5±9.6	77.7±11.6	0.66
RR interval	962.5±11.7	961.8±14.1	963.0±9.8	0.31
PR interval	168.2±14.7	165.1±15.8	170.4±13.4	<0.001
QRS	103.1±11.9	103.2±11.6	103.0±12.2	0.90
QT	391.7±30.7	391.7±27.5	391.8±32.8	0.31
QTc	443.3±21.5	442.5±20.0	443.8±22.5	0.66
**Echocardiography**				
AOD	33.3±3.4	33.5±3.2	33.1±3.5	0.03
LAD	46.3±6.3	44.6±6.1	47.4±6.3	<0.001
LVDd	49.2±3.9	49.4±4.0	49.1±3.9	0.32
LVDs	32.2±3.0	32.4±3.1	32.0±3.0	0.10
IVST	10.1±1.6	10.1±1.5	10.1±1.7	0.66
FS	34.7±3.4	34.4±2.7	35.0±3.9	0.04
SV	72.5±13.0	72.9±13.5	72.3±12.6	0.69
LVEF	63.3±4.0	63.0±4.2	63.5±3.8	0.08
LAAFV	55.3±15.3	57.8±14.1	53.5±15.9	<0.001

ACEI: angiotensin-converting enzyme inhibitors; AF: atrial fibrillation; AOD: aortic diameter; ARBs: angiotensin II receptor blockers; ARNIs: angiotensin receptor-neprilysin inhibitors; BMI: body mass index; BNP: brain natriuretic peptide; CAD: coronary artery disease; DBP: diastolic blood pressure; FS: fractional shortening; Hb: hemoglobin; HDLC: high-density lipoprotein cholesterol; HR: heart rate; IVST: interventricular septum thickness; LAAFV: left atrial appendage flow velocity; LAD: left atrial diameter; LDLC: low-density lipoprotein cholesterol; LVDd: left ventricular diastolic diameter; LVDs: left ventricular systolic diameter; LVEF: left ventricular ejection fraction; PCI: percutaneous coronary intervention; PFO: patent foramen ovale; PR interval: interval between the onset of the P-wave and the start of the QRS complex; QRS: QRS (ventricular depolarization); QT: total time of ventricular depolarization and repolarization; QTc: corrected QT interval; RBC: red blood cell; RR interval: interval between consecutive R waves; SBP: systolic blood pressure; ST2: suppression of tumorigenicity 2; SV: stroke volume; TIA: transient ischemic attack; WBC: white blood cell.

Laboratory analyses revealed significantly higher BNP levels in the recurrence group (458.2 ± 277.2 vs. 285.7 ± 210.5 pg/mL, P < 0.001), whereas other laboratory parameters remained comparable between the groups. In terms of medication usage, patients in the recurrence group were less frequently prescribed statin therapy (25% vs. 41%, P < 0.001), rivaroxaban (64% vs. 75%, P < 0.01), and ARNIs (4% vs. 8%, P = 0.04).

Electrocardiographic analysis showed significantly prolonged PR intervals in the recurrence group (170.4 ± 13.4 vs. 165.1 ± 15.8 ms, P < 0.001). Echocardiographic parameters differed across the groups, with the recurrence group demonstrating a larger LAD (47.4 ± 6.3 vs. 44.6 ± 6.1 mm, P < 0.001), a reduced LAAFV (53.5 ± 15.9 vs. 57.8 ± 14.1 cm/s, P < 0.001), a smaller aortic diameter (33.1 ± 3.5 vs. 33.5 ± 3.2 mm, P = 0.03), and greater fractional shortening (35.0 ± 3.9% vs. 34.4 ± 2.7%, P = 0.04).

To ensure robust model development and validation, the samples were randomly divided into training (531 patients, 58.8% recurrence) and validation (226 patients, 60.2% recurrence) sets at a 70%/30% split. Table 2 shows that there were no notable differences in baseline characteristics between the two groups, confirming their comparability for later analyses.

**Table 2:** Clinical and echocardiographic characteristics of the training and validation sets *of* atrial fibrillation patients.

**Characteristic**	**Total** **(n = 757)**	**Training set** **(n = 531)**	**Validation set (n = 226)**	***P* value**
Age (years)	60.65±10.61	61.10±10.50	59.60±10.82	0.06
Female [n (%)]	306 (40)	213 (40)	93 (41)	0.85
BMI(kg/m^2^)	25.31±3.31	25.40±3.36	25.10±3.18	0.56
persistent AF [n (%)]	239 (32)	167 (31)	72 (32)	0.98
AF History(months)	26.20±42.93	28.16 ± 43.04	21.57 ± 42.40	0.06
CHA_2_DS_2_-VASc	1.67±1.40	1.73 ± 1.41	1.52 ± 1.36	0.06
SBP (mmHg)	129.14±13.30	129.06±13.40	129.32±13.08	0.98
DBP (mmHg)	76.28±8.95	75.87 ± 8.94	77.26 ± 8.92	0.07
**Comorbidities [n (%)]**				
Hypertension	364 (52.1)	267 (54.4)	97 (46.6)	0.07
Diabetes	99 (14.2)	71 (14.5)	28 (13.5)	0.82
Stroke/TIA	72 (10.3)	51 (10.4)	21 (10.1)	1.00
PFO	11 (1.6)	7 (1.4)	4 (1.9)	0.74
PCI [n (%)]	29 (4.1)	21 (4.3)	8 (3.8)	0.96
Smoking	142 (20.3)	93 (18.9)	49 (23.6)	0.20
Alcohol	108 (15.5)	74 (15.1)	34 (16.3)	0.76
CAD	88 (12.6)	57 (11.6)	31 (14.9)	0.28
**Laboratory data**				
WBC	5.91±1.52	5.87±1.49	6.02±1.59	0.18
RBC	4.44 ± 0.52	4.44 ± 0.53	4.45 ± 0.5	0.86
Hb(g/L)	135.01±16.35	134.93±15.71	135.2±17.81	0.84
Creatinine (µmol/L)	71.86±12.44	72.10±12.91	71.28±11.25	0.16
Urea (mmol/L)	5.68±1.56	5.72±1.53	5.59±1.61	0.19
Uric acid (µmol/L)	324.78±97.12	323.33±98.38	328.20±94.22	0.58
Total cholesterol (mmol/L)	4.21±0.98	4.21±0.97	4.22±0.99	0.66
Triglyceride (mmol/L)	1.61±1.04	1.62±1.08	1.58±0.94	0.82
HDL-C (mmol/L)	1.09±0.26	1.09±0.26	1.08±0.27	0.76
LDL-C (mmol/L)	2.42±0.78	2.41±0.77	2.44±0.78	0.59
ST2 (ng/mL)	20.30±23.06	19.74±23.57	21.63±21.81	0.05
CKMB	42.05±31.79	42.40±32.66	41.22±29.71	0.78
BNP(pg/mL)	449.46±888.21	462.58±932.58	418.48±774.68	0.93
**Medications [n (%)]**				
Propafenone	158 (22.6)	110 (22.4)	48 (23.1)	0.92
Amiodarone	268 (38.3)	187 (38.1)	81 (38.9)	0.90
Dronedarone	37 (5.3)	25 (5.1)	12 (5.8)	0.86
Wuxin particle	40 (5.7)	25 (5.1)	15 (7.2)	0.35
Beta-blockers	229 (32.8)	166 (33.8)	63 (30.3)	0.41
Anticoagulants	672 (96.1)	473 (96.3)	199 (95.7)	0.84
Warfarin	21 (3.0)	16 (3.3)	5 (2.4)	0.72
Rivaroxaban	260 (37.2)	190 (38.7)	70 (33.7)	0.23
Dabigatran	213 (30.5)	159 (32.4)	54 (26.0)	0.11
Statins	273 (39.1)	195 (39.7)	78 (37.5)	0.64
ARNIs	42 (6.0)	27 (5.5)	15 (7.2)	0.49
ACEI	36 (5.2)	26 (5.3)	10 (4.8)	0.94
ARBs	180 (25.8)	131 (26.7)	49 (23.6)	0.44
Loop diuretics	13 (1.9)	6 (1.2)	7 (3.4)	0.11
Thiazide diuretics	30 (4.3)	25 (5.1)	5 (2.4)	0.16
Spironolactone	20 (2.9)	10 (2.0)	10 (4.8)	0.08
**Electrocardiograph**				
HR	78.65±27.08	79.49±31.59	76.67±10.20	0.07
RR interval	767.83±64.73	766.10±66.68	771.93±59.81	0.21
PR interval	162.84±13.80	162.69±13.79	163.17±13.86	0.65
QRS	104.21±11.81	103.94±11.82	104.86±11.80	0.09
QT	390.90±32.70	390.09±32.68	392.82±32.76	0.48
QTc	449.19±143.65	451.85±170.78	442.91±21.91	0.19
**Echocardiography**				
AOD	33.25 ± 3.38	33.13 ± 3.45	33.52 ± 3.2	0.05
LAD	43.39±4.91	43.34±4.98	43.51±4.77	0.68
LVDd	49.07±4.29	48.95±4.39	49.35±4.05	0.33
LVDs	32.11±3.08	32.00±2.99	32.37±3.27	0.38
IVST	10.04±1.66	10.08±1.71	9.95±1.54	0.33
FS	9.17±1.07	9.18±1.09	9.16±1.02	0.70
SV	34.83±3.37	34.98±3.62	34.47±2.68	0.11
LVEF	72.41±12.91	72.33±12.59	72.60±13.68	0.67
LAAFV	55.48±14.69	56.06±15.05	54.12±13.73	0.11

ACEI: angiotensin-converting enzyme inhibitors; AF: atrial fibrillation; AOD: aortic diameter; ARBs: angiotensin II receptor blockers; ARNIs: angiotensin receptor-neprilysin inhibitors; BMI: body mass index; BNP: brain natriuretic peptide; CKMB: creatine kinase-myocardial band; DBP: diastolic blood pressure; FS: fractional shortening; Hb: hemoglobin; HDLC: high-density lipoprotein cholesterol; HR: heart rate; IVST: interventricular septum thickness; LAAFV: left atrial appendage flow velocity; LAD: left atrial diameter; LDLC: low-density lipoprotein cholesterol; LVDd: left ventricular diastolic diameter; LVDs: left ventricular systolic diameter; LVEF: left ventricular ejection fraction; PCI: percutaneous coronary intervention; PFO: patent foramen ovale; PR interval: interval between the onset of the P-wave and the start of the QRS complex; QRS: QRS complex (ventricular depolarization); QT: total time of ventricular depolarization and repolarization; QTc: corrected QT interval; RBC: red blood cell; RR interval: interval between consecutive R waves; SBP: systolic blood pressure; ST2: suppression of tumorigenicity 2; SV: stroke volume;TIA: transient ischemic attack; WBC: white blood cell.

### Predictors of AF recurrence

**Fig. 2:**
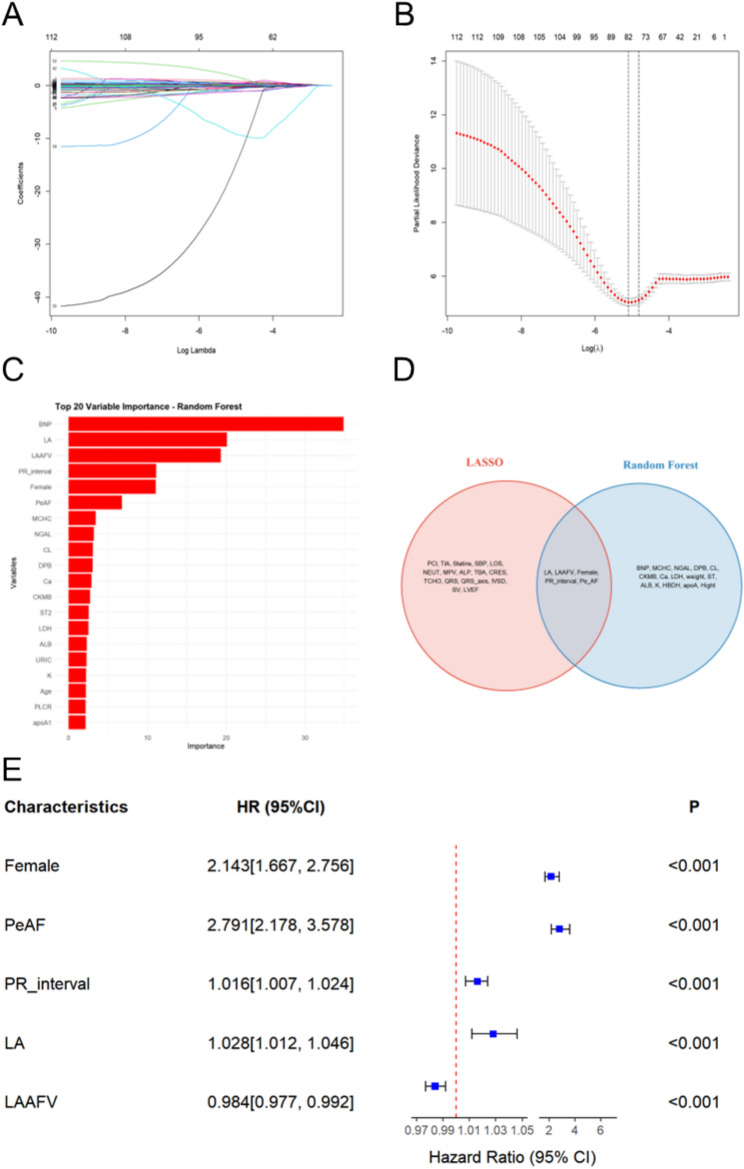
Identification for factors affecting AF recurrence after cryoballoon ablation. (**A**) LASSO regression coefficient profiles of variables against log(lambda). (**B**) Tuning parameter (lambda) selection in LASSO regression via 10-fold cross-validation. The left vertical line denotes the lambda value with minimum cross-validation error, whereas the right vertical line represents lambda within one standard error of the minimum. (**C**) Top 20 variables ranked by variable importance via the random forest algorithm. (**D**) Venn diagram showing the intersection of variables selected by the LASSO and random forest methods. (**E**) Multivariate Cox regression analysis of the selected variables showing hazard ratios with 95% confidence intervals. LASSO, least absolute shrinkage and selection operator; LA, left atrial dimension; LAAFV, left atrial appendage flow velocity; PeAF, persistent atrial fibrillation; PRinterval, PR interval on electrocardiogram.

The study began by evaluating 124 candidate variables for possible inclusion in the prediction model. Using LASSO regression and a 10-fold cross-validation, 21 variables were identified as candidate predictors (Figure 2A, 2B). These included persistent AF, female sex, percutaneous coronary intervention (PCI), transient ischemic attack (TIA), statins, systolic blood pressure (SBP), length of hospital stay (LOS), neutrophil count (NEUT), mean platelet volume (MPV), alkaline phosphatase (ALP), total bile acid (TBA), serum creatinine (Cr, total cholesterol (TC), the PR interval, QRS duration (QRS), QRS axis, LAAFV, LAD, interventricular septal thickness (IVST), stroke volume (SV), and left ventricular ejection fraction (LVEF).

Random forest analysis was performed as an alternative feature-selection method. The top 20 variables ranked by variable importance were identified (Figure 2C), which included BNP, LAD, LAAFV, the PR interval, female sex, PeAF, mean corpuscular hemoglobin concentration (MCHC), neutrophil gelatinase-associated lipocalin (NGAL), chloride (CL), diastolic blood pressure (DBP), calcium (Ca), creatine kinase-MB (CKMB), soluble growth stimulation expressed gene 2 (sST2), lactate dehydrogenase (LDH), albumin (ALB), uric acid (URIC), potassium (K), age, platelet large cell ratio (PLCR), and apolipoprotein A1 (apoA1). We subsequently performed an overlap analysis on the variables identified by both the LASSO regression and random forest methods to identify common predictors that demonstrated significant importance in both approaches. This analysis revealed five overlapping variables: LAD, LAAFV, female sex, PR interval, and PeAF (Figure 2D). The identification of these common variables via two distinct feature selection methods increased our confidence in their predictive ability and informed subsequent model construction.

Multivariate Cox regression analysis was performed on these five overlapping variables to evaluate their independent prognostic value for AF recurrence (Figure 2E). The results demonstrated that persistent AF was associated with the highest risk of recurrence (HR 2.791, 95% CI 2.178–3.578; P<0.001), followed by female sex (HR 2.143, 95% CI 1.667–2.756; P<0.001). Among the cardiac parameters, increased LAD (HR 1.028, 95% CI 1.012–1.046, P<0.001), prolonged PR interval (HR 1.016, 95% CI 1.007–1.024, P<0.001), and decreased LAAFV (HR 0.984, 95% CI 0.977–0.992, P<0.001) were independently associated with AF recurrence. These findings indicate that both clinical characteristics and cardiac structural/functional parameters are robust independent predictors of AF recurrence. Notably, the combination of these variables provides a comprehensive risk assessment framework that incorporates both the anatomical and physiological factors that influence AF recurrence.

### Development of a nomogram for AF recurrence

**Fig. 3:**
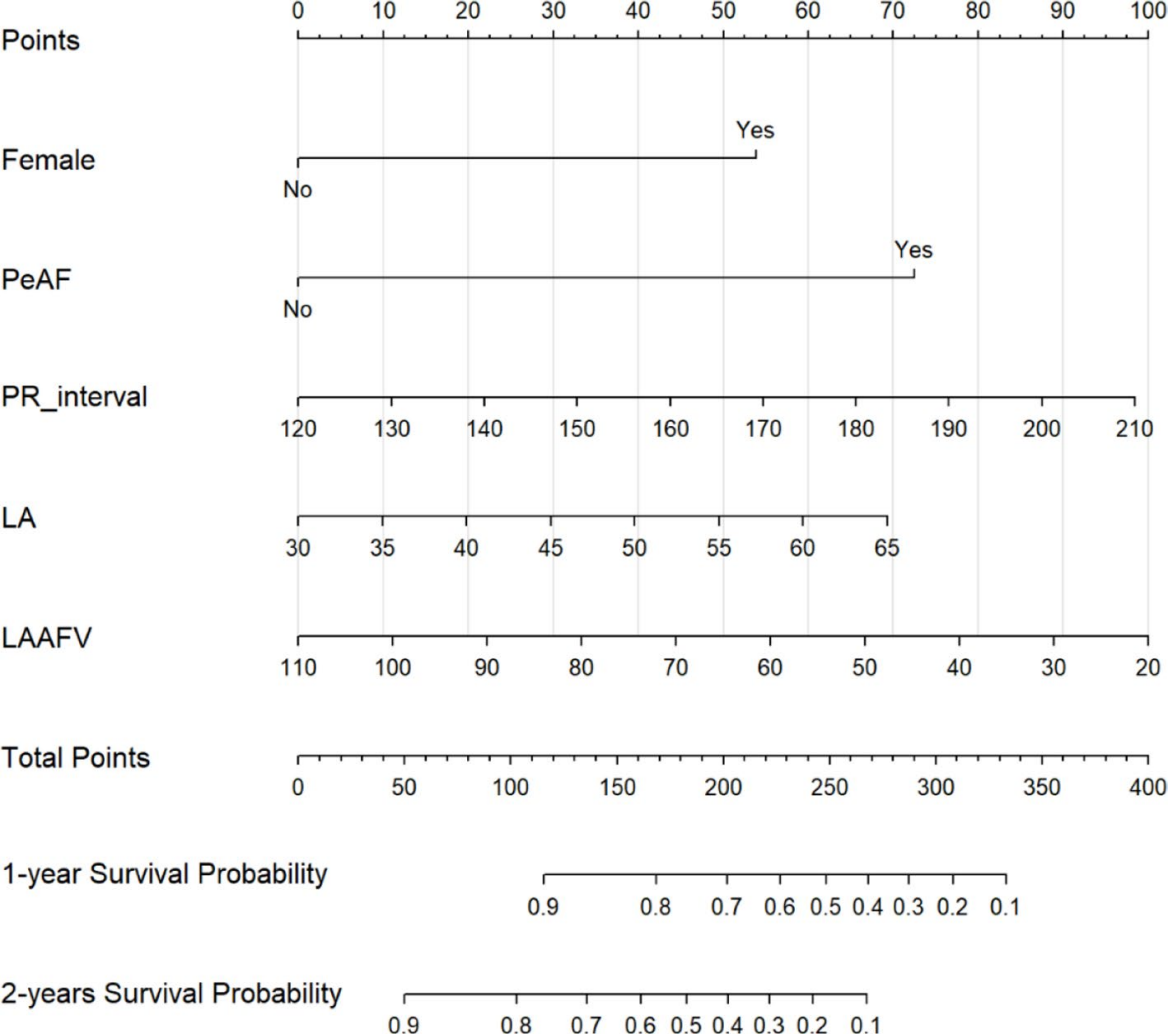
Nomogram for predicting AF recurrence after cryoballoon ablation. The nomogram integrates key predictors, including female sex, persistent atrial fibrillation (PeAF), the PR interval, the left atrial diameter (LA), and the left atrial appendage flow velocity (LAAFV). The total points derived from these predictors correspond to the 1-year and 2-year survival probabilities shown at the bottom of the nomogram.

On the basis of the five independent predictors identified in the multivariate Cox analysis, we constructed a nomogram to estimate the individualized probabilities of AF-free survival at 12 and 24 months after cryoballoon ablation (Figure 3). In this nomogram, each predictor was assigned a corresponding point value on a scale of 0-100, with persistent AF and female sex contributing the highest maximum points. The cumulative points, which were calculated by summing the individual scores for each predictor (ranging from 0-400), were mapped to predict the probability of AF-free survival. The total points are inversely correlated with survival probability, where higher scores indicate lower AF-free survival rates at both the 1- and 2-year time points. This visual predictive tool enables clinicians to perform individualized risk stratification and potentially optimize postablation management strategies.

**Fig. 4:**
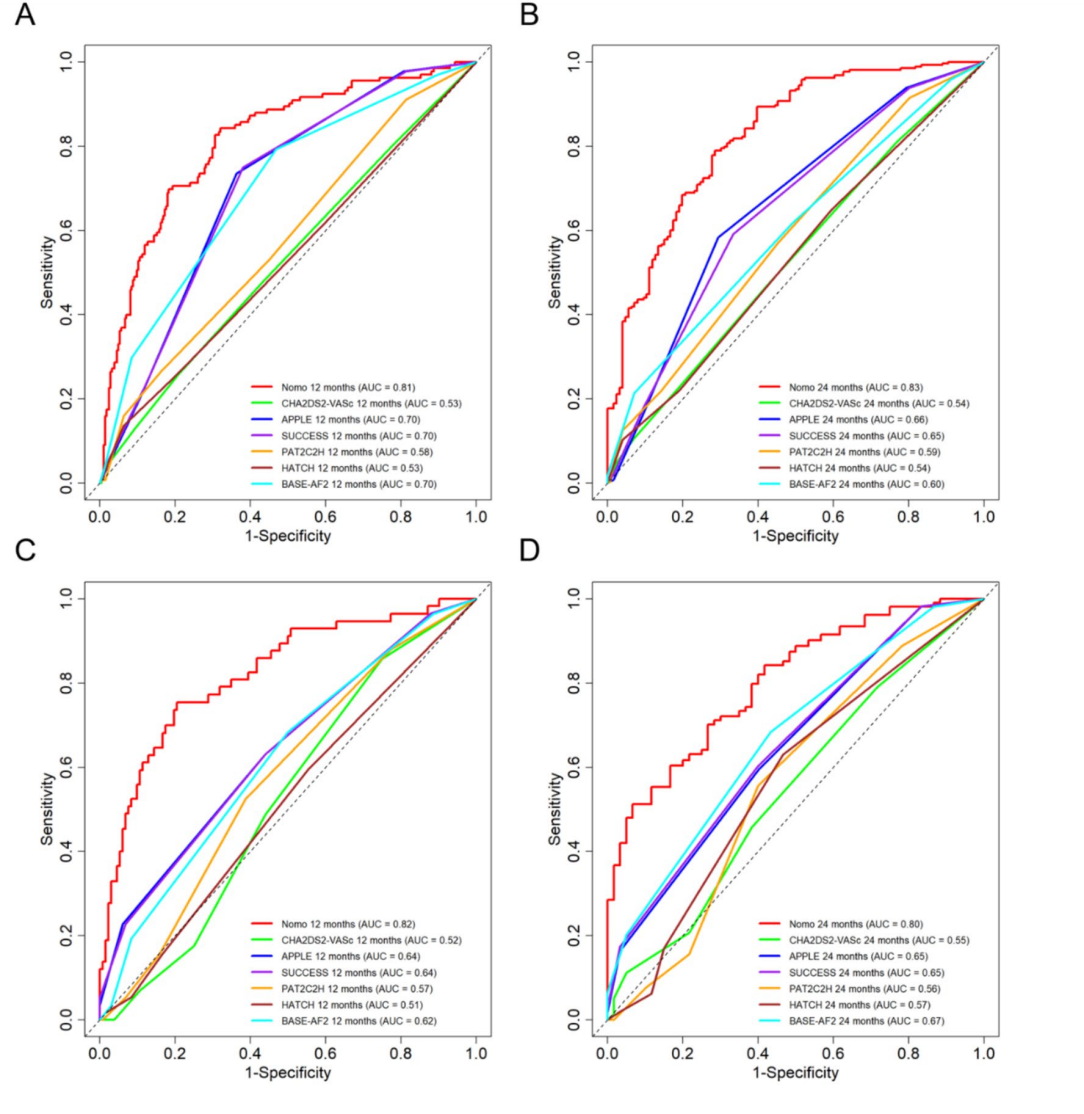
Receiver operating characteristic (ROC) curves: discrimination of the proposed nomogram vs. established AF recurrence scores at 12 and 24 months. (**A**) Training cohort, 12 months; (**B**) Validation cohort, 12 months; (**C**) Training cohort, 24 months; (**D**) Validation cohort, 24 months. The proposed model (red) is plotted against APPLE, SUCCESS, PAT2C2H, HATCH, BASE-AF2, and CHA₂DS₂-VASc using the same participants at each horizon (participants with ≥horizon follow-up or an event before that horizon). AUC point estimates appear in the legends. 95% confidence intervals for the AUC were obtained via nonparametric bootstrap (B = 1000). Two-sided, paired DeLong tests were used for pairwise comparisons against the proposed model, with Holm adjustment for multiplicity within cohorts and horizons; adjusted P values are reported in Supplementary Tables S1–S2. The diagonal dashed line denotes no-discrimination (AUC = 0.5).

### Model performance and validation

ROC curves were used to assess the discriminatory ability of the model. In the training cohort, the model demonstrated strong predictive ability, with area under the curve (AUCs) of 0.81 (95% CI: 0.77-0.86) and 0.83 (95% CI: 0.78-0.87) for 1- and 2-year AF recurrence prediction, respectively (Figure 4A and 4 C, Supplementary Table S1). The model’s robustness was further confirmed in the validation cohort, which showed comparable discriminative performance, with AUC values of 0.82 (95% CI: 0.75–0.89) at 12 months and 0.80 (95% CI: 0.73–0.87) at 24 months (Figure 4B and 4D, Supplementary Table S2). The consistency of these results between the training and validation cohorts, with all AUC values exceeding 0.80, demonstrates the excellent discriminative ability and reliability of the model for predicting AF recurrence after cryoballoon ablation.

**Fig. 5:**
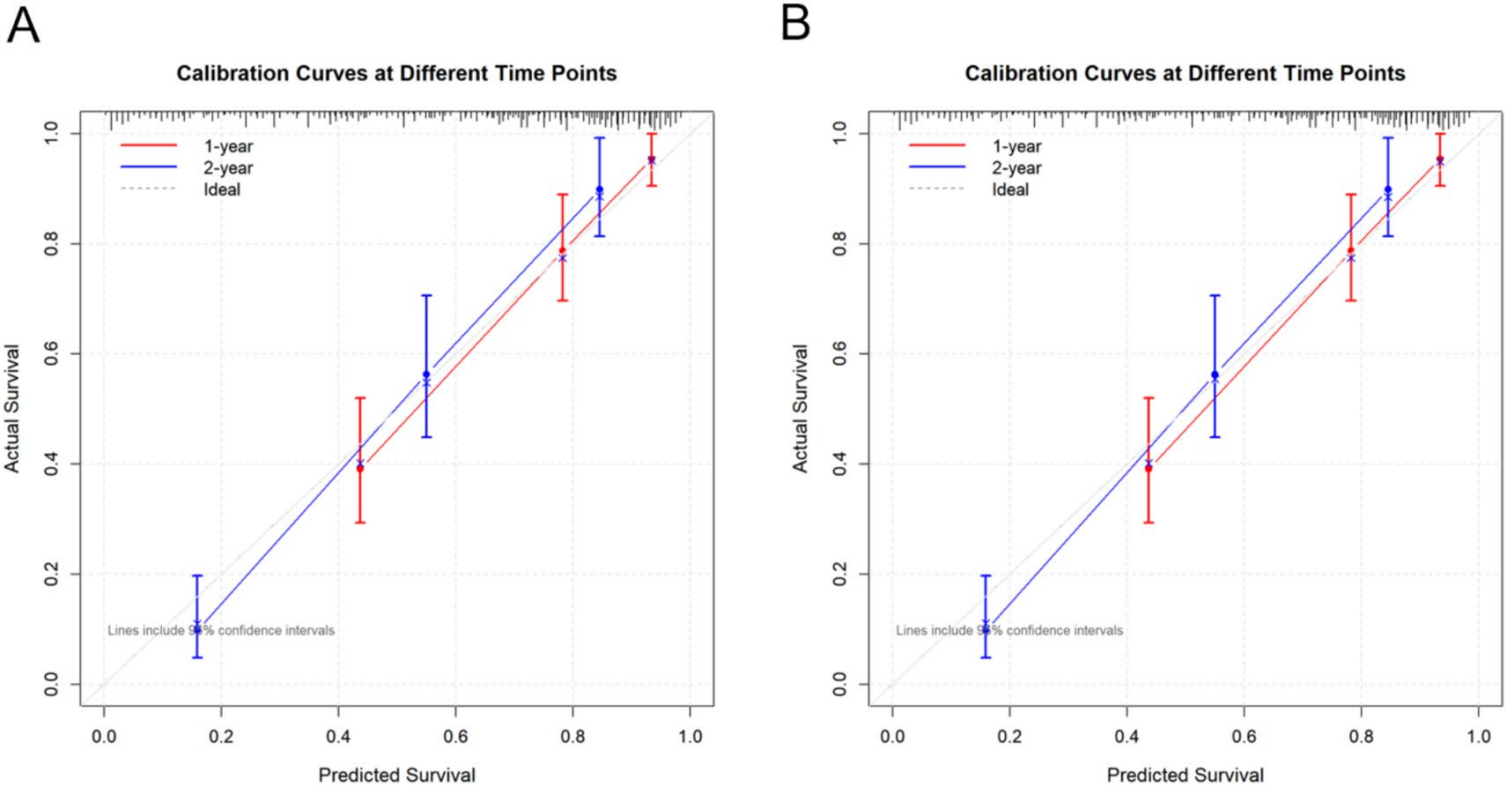
Calibration performance evaluation of the predictive model for AF recurrence after cryoballoon ablation in the training and validation sets. (**A**) Calibration curves for 1- and 2-year AF-free recurrence prediction in the training set. **(B)** Calibration curves for 1- and 2-year AF-free recurrence prediction in the validation set. CI, confidence interval; AF, atrial fibrillation.

The calibration plots (Figures 5A and 5B) revealed a strong correlation between the predicted and actual recurrence rates. The calibration curves for the 1-year and 2-year forecasts in both the training and validation cohorts closely followed the ideal line, with most data points within the 95% confidence intervals. The calibration performance was particularly robust in the middle-risk range (predicted survival 0.4–0.8), suggesting the reliable risk stratification capability of the nomogram.

**Fig. 6:**
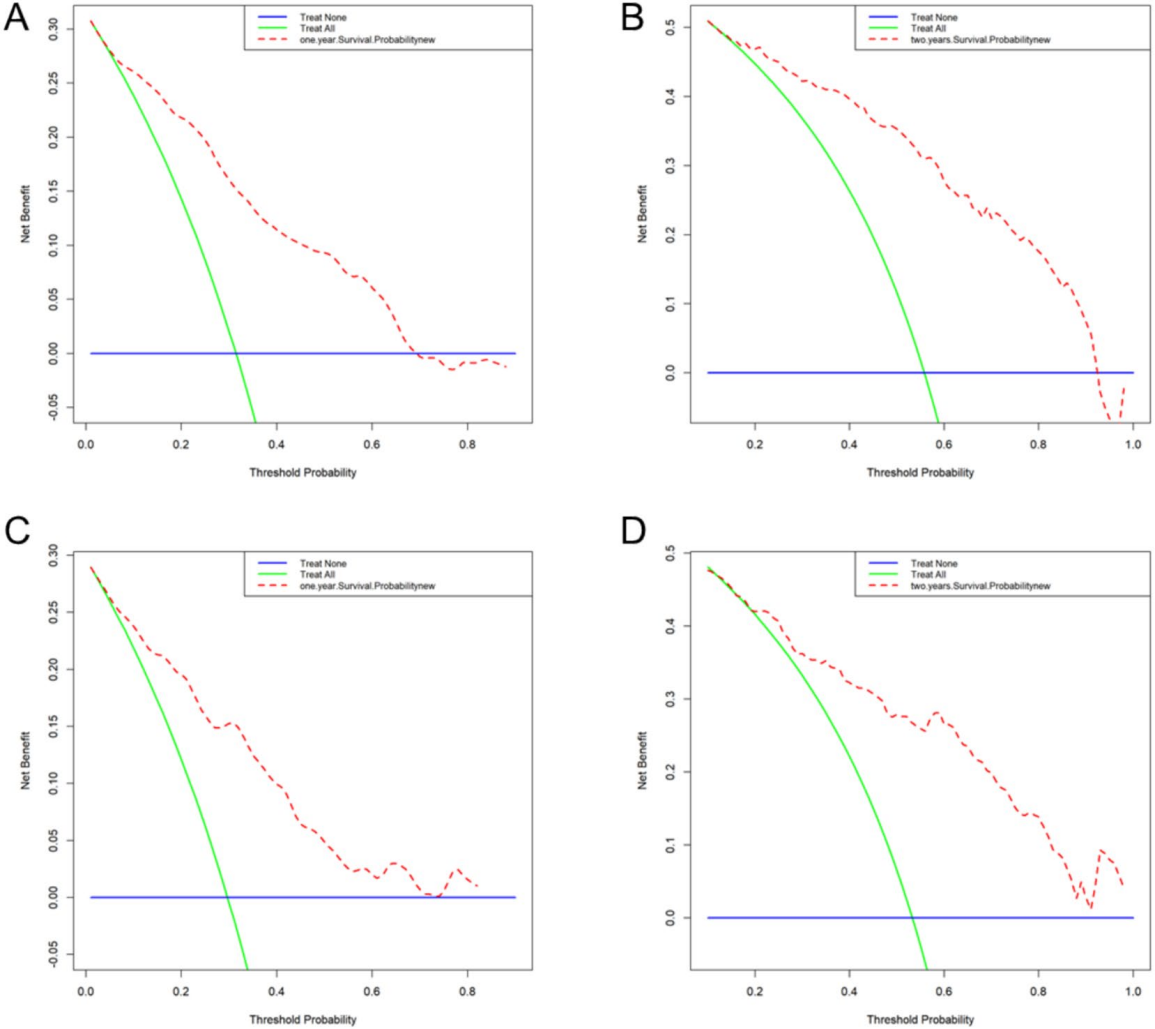
Clinical utility assessment via decision curve analysis (DCA) for the predictive model. (**A**) DCA for 12-month AF recurrence risk prediction in the training set. (**B**) DCA for 24-month AF recurrence risk prediction in the training set. (**C**) DCA for 12-month AF recurrence risk prediction in the validation set. (**D**) DCA for 24-month AF recurrence risk prediction in the validation set.

Decision curve analysis (DCA) revealed that the nomogram demonstrated potential clinical utility in both the training and validation groups (Figure 6A-6D). The model demonstrated positive net benefits for 12- and 24-month forecasts over a broad spectrum of threshold probabilities (0-80%) (Figures 6A-6D). The nomogram consistently outperformed both the “treat all” and “treat none” strategies, with particularly robust performance in the threshold probability range of 0-40%. The similar DCA curves in both the training and validation groups further validated the model’s consistent predictive value and dependable clinical use in predicting short- and long-term outcomes.

### Risk stratification and predictive analysis

**Fig. 7:**
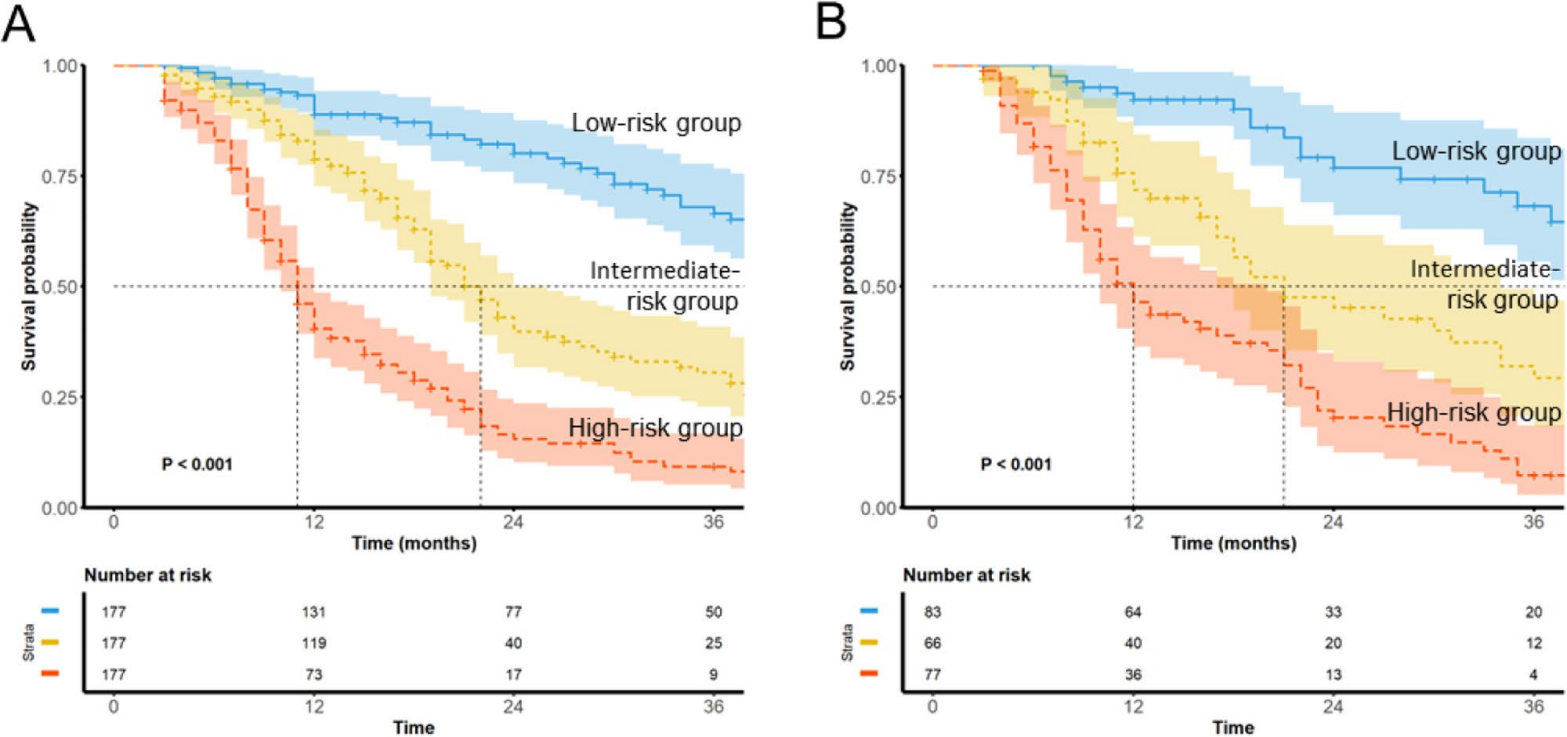
Risk stratification for AF recurrence after cryoballoon ablation via nomogram-based scores (**A**) Kaplan‒Meier curves showing AF-free survival in the training cohort (n = 531) stratified into low-risk (n = 177), intermediate-risk (n = 177), and high-risk (n = 177) groups. The median follow-up duration was 36 months approximately, with significant survival differences among the groups (log-rank P<0.001). (**B**) Validation of risk stratification in the validation cohort (n = 226), showing consistent risk separation among the low-risk (n = 83), intermediate-risk (n = 66), and high-risk (n = 77) groups (log-rank P<0.001). The shaded areas represent 95% confidence intervals. The numbers at risk are shown below each plot.

On the basis of the tertiles of nomogram total point scores derived from the training cohort (cutoff values: 160 and 217), patients were categorized into three risk groups: low-risk (≤ 160 points, n = 177), intermediate-risk (161–217 points, n = 177), and high-risk (> 217 points, n = 177). Across the risk groups, there was a progressive increase in recurrence -free survival across both the training and validation cohorts. At 24 months, the recurrence-free survival rates were approximately 80%, 45%, and 20% for the low-, intermediate-, and high-risk groups, respectively, in the training cohort, with similar stratification rates observed in the validation cohort (75%, 50%, and 25%, respectively) (Figure 7A).

Kaplan–Meier survival analysis demonstrated significant differences among the three risk groups (log-rank P<0.001 in both cohorts) (Figure 7B). The survival curves exhibited early separation within the first 12 months and maintained distinct trajectories throughout the 36-month follow-up period. This risk stratification was consistently validated in the validation cohort, confirming the ability of the nomogram to identify patients with differential recurrence risk effectively. The similar pattern and magnitude of separation between the risk groups in both cohorts further supported the reliability and reproducibility of this risk stratification system.

In the training cohort, the nomogram achieved AUCs of 0.81 (12 months) and 0.83 (24 months), exceeding those of CHA₂DS₂-VASc (0.53, 0.54), APPLE (0.70, 0.66), SUCCESS (0.70, 0.65), PAT2C2H (0.58, 0.59), HATCH (0.53, 0.54), and BASE-AF2 (0.70, 0.60); paired DeLong tests with Holm adjustment confirmed superiority at both horizons (Supplementary Table S1; Figure 4A–B). In the validation cohort, the AUCs were 0.82 (12 months) and 0.80 (24 months), again surpassing APPLE (0.64, 0.65), SUCCESS (0.64, 0.65), PAT2C2H (0.57, 0.56), HATCH (0.51, 0.57), BASE-AF2 (0.62, 0.67), and CHA₂DS₂-VASc (0.52, 0.55); all Holm-adjusted paired comparisons were significant (Supplementary Table S2; Figure 4C–D). Overall, the nomogram provided the best discrimination among all tools at both 12 and 24 months, with consistent risk separation across cohorts.

## Discussion

This study developed and validated a risk score for predicting AF recurrence after cryoballoon ablation. Five predictors—female sex, persistent AF, prolonged PR interval, larger LAD, and lower LAAFV—were independently associated with recurren mitigate the risk of spectrum or setting biasce, which is consistent with the atrial substrate and conduction pathophysiology. Few cryoablation-focused models have incorporated electrocardiographic indices; here, the inclusion of the PR interval underscores the contribution of atrial conduction abnormalities to the risk of recurrence. The score demonstrated strong discrimination and clinically meaningful risk stratification, with early and sustained separation of Kaplan–Meier curves and the higher absolute event rates in the high-risk stratum. To contextualize performance, we prespecified head-to-head benchmarking at 12 and 24 months: the nomogram achieved AUCs of 0.81/0.83 in the training cohort and 0.82/0.80 in the validation cohort and outperformed APPLE, SUCCESS, PAT2C2H, HATCH, BASE-AF2, and CHA₂DS₂-VASc.

This model incorporates novel predictors, such as the PR interval and LAAFV, expanding beyond the clinical parameters traditionally included in models, such as the CHADS_2_ and CHA_2_DS_2_-VASc scores^3,16^. The PR interval reflects the atrial conduction time and provides a unique electrocardiographic perspective on delayed atrial conduction, a factor often overlooked in previous models^17,18^. Similarly, LAAFV represents a dynamic echocardiographic marker of left atrial function, offering insights into the mechanical and thrombogenic risks that LAD alone cannot provide^16,19^. By integrating measures of atrial substrate (LAD), conduction (PR interval), and mechanical function (LAAFV), the nomogram addresses complementary pathophysiological mechanisms that are likely to contribute to postablation recurrence.

Compared with established ablation-focused scores (APPLE, SUCCESS, PAT2C2H, HATCH, BASE-AF2) and CHA₂DS₂-VASc, our model demonstrated superior discrimination at both 12 and 24 months in the same cohort, with all pairwise comparisons showing statistical significance after Holm correction (Tables S1–S2). These findings contextualize the model’s performance within contemporary cryoablation practice and mitigate the risk of spectrum or setting bias that may arise from evaluating models in isolation. Furthermore, decision curve analysis and calibration supported the model’s clinical utility and reliability across both time horizons.

A prolonged PR interval indicates delayed atrial conduction, potentially forming a basis for re-entrant circuits and increasing the likelihood of AF recurrence^13,14^. This delay is linked to electroanatomical remodeling, which includes low-voltage regions in the left atrium that promote reentrant circuit formation and structural alterations that contribute to AF pathophysiology^20^. Furthermore, Mendelian randomization studies suggested a causal link between prolonged PR intervals and recurrence risk after cryoballoon, highlighting the genetic predisposition to AF^21^. Together, these findings support the inclusion of the PR interval as a mechanistically plausible and empirically validated predictor of AF recurrence in ablation cohorts.

Meta-analyses reinforce the significance of the PR interval as an independent risk factor for AF, challenging the traditional perception of first-degree AV blocks as benign^22^. In specific populations, such as individuals with repaired tetralogy of Fallot, prolonged PR intervals reliably predict atrial tachyarrhythmias, establishing their utility as simple yet robust predictors^23^. Findings from the LIFE Adult Study further support this, revealing that patients with prolonged PR intervals share echocardiographic and biomarker profiles with AF patients, positioning PR interval prolongation as a precursor to arrhythmias^24^. In our study, the PR interval remained independently significant after adjusting for LAD and LAAFV, indicating that it provides additive prognostic information rather than being redundant with structural or functional markers.

The LAAFV is a pivotal echocardiographic parameter for assessing the risk of AF recurrence. Research, including systematic reviews and meta-analyses, has indicated that a decrease in LAAFV is significantly correlated with higher rates of recurrence after catheter ablation. Increasing the LAAFV by 1 cm/s decreases the chance of recurrence by approximately 3%, underscoring its prognostic significance^15^. A nonlinear connection between the peak flow velocity of the left atrial appendage (LAA) and AF recurrence has been noted, with reduced LAAFV especially associated with increased recurrence rates in female patients^25^. A low LAAFV reflects impaired mechanical function of the LAA, contributing to blood stasis, thrombus formation, and elevated recurrence rates^26,27^. Studies suggest that incorporating LAAFV into a multifaceted evaluation of left atrial function enhances risk stratification and clinical management^28,29^. Importantly, LAAFV provides a dynamic and functional perspective on atrial appendage hemodynamics, complementing static markers, such as the LAD. Our findings further support this finding by demonstrating that LAAFV improves discrimination in cryoablation populations when evaluated alongside the PR interval and LAD.

Integrating LAAFV into predictive models represents an important extension, enabling more precise stratification and guiding targeted interventions. Strategies such as optimizing anticoagulation therapy or employing ablation techniques that improve LAA function could potentially influence recurrence risk and improve patient outcomes. Although our study was not designed to assess management strategies based on LAAFV or PR intervals, the observed risk gradients provide a strong rationale for the prospective evaluation of such personalized approaches.

Although the score was derived and internally validated in a cryoballoon ablation cohort, its predictors—female sex, persistent AF, prolonged PR interval, larger LAD, and reduced LAAFV—index atrial substrate and conduction rather than energy-source–specific effects. This biological focus suggests potential transportability to other ablation modalities, including radiofrequency and pulsed-field ablation. Even so, differences in lesion durability and reconnection patterns across technologies may shift the baseline hazard and degrade calibration. Accordingly, application to RF or PFA populations should follow external validation with recalibration (calibration-in-the-large and calibration slope). If technology materially modifies predictor effects, model updating that adds a technology indicator and prespecified interaction terms is warranted. A prespecified plan for external validation, recalibration, and model updating is provided in the Supplement. Future studies should also explore whether model-based surveillance (e.g., intensified rhythm monitoring for high-risk patients) can improve clinical outcomes and optimize resource utilization.

Despite its strengths, this study has several limitations. First, this was a single-center retrospective study. Although we employed a split-sample approach for internal validation, the lack of an external cohort from a different institution limits the generalizability of our findings to populations with different demographics or operator experiences. Second, postablation monitoring relied on intermittent 24-hour Holter and ECGs rather than continuous monitoring (e.g., implantable loop recorders). While this inevitably underestimates the true recurrence rate-particularly of asymptomatic episodes-this detection bias likely affects all risk strata similarly and thus may not materially alter the relative discriminatory performance of the model. Third, LAAFV measurement via transesophageal echocardiography is inherently operator-dependent. Although measurements were performed by experienced sonographers in our center, inter-observer variability remains a potential source of error that necessitates standardized acquisition protocols for broader reproducibility. Fourth, regarding benchmarking, we acknowledge that the performance of some established scores might have been underestimated because specific predictors (e.g., obstructive sleep apnea parameters for PAT2C2H) were not routinely collected in our historical cohort and thus could not be fully reconstructed. Finally, residual confounding remains possible despite multivariable adjustment, as unmeasured factors such as genetic predisposition or detailed atrial fibrosis burden (e.g., MRI-based fibrosis quantification) were not included. Future studies with rigorous external validation and standardized protocols are required to address these limitations.

Beyond the variables retained in our model, prior work indicates that serum biomarkers—excluding natriuretic peptides per our analytic focus—such as high-sensitivity troponin and inflammation/fibrosis markers (hs-CRP, galectin-3, GDF-15, and sST2), as well as quantitative indices of AF electrical complexity (e.g., low-voltage area, fractionated/complex electrograms, AF cycle length/dominant frequency, and entropy), may enhance discrimination^30,31^. In this cohort, these biomarkers and mapping-based complexity measures were not uniformly collected or standardized and were therefore excluded to minimize measurement heterogeneity and selection bias. Future studies should standardize the acquisition of these domains and quantify their incremental value over the current model via prespecified metrics—such as net reclassification improvement, integrated discrimination improvement—and decision curve analysis. In addition, prospective multicenter studies with extended follow-up, larger and more diverse samples, and head-to-head external benchmarking against established cryoablation scores via standardized ECG and echocardiographic protocols (particularly for LAAFV) are warranted.

We developed and internally validated a risk score integrating clinical characteristics with atrial substrate and functional indices (PR interval, LAD, and LAAFV) within a single-center cryoballoon cohort. In this study population, the score exhibited higher discriminatory accuracy at both 12 and 24 months compared with CHA₂DS₂-VASc, APPLE, SUCCESS, PAT2C2H, HATCH, and BASE-AF2, as confirmed by paired DeLong tests with Holm correction. This tool may facilitate risk stratification to guide surveillance and postablation management. While the predictors likely reflect general atrial remodeling, application to radiofrequency (RF) or pulsed-field ablation (PFA) populations requires specific external validation with recalibration (including calibration-in-the-large and slope) and potential model updating. Multicenter studies with longer follow-up times, standardized LAAFV measurements, and assessments of clinical impact are warranted to confirm these findings.

## Materials and methods

### Inclusion and exclusion criteria

This retrospective analysis included 757 patients with atrial fibrillation (AF) who underwent first-time cryoballoon ablation at the Cardiology Department of Nanjing First Hospital between January 2017 and December 2023. The eligibility criteria included a verified AF diagnosis via medical history, ECG, or Holter monitoring, along with the completion of a cryoballoon ablation procedure. The exclusion criteria were as follows: (1) history of AF ablation, (2) end-stage renal failure, (3) thrombus in the left atrium or its appendage, (4) valvular AF, and (5) noncompliance or refusal to take postoperative oral anticoagulant (OAC) therapy. Eligibility was applied a priori and independent of outcomes; patients were not selected according to the AF recurrence status. Of the 886 procedures screened, 55 were excluded for incomplete echocardiographic/laboratory data, and 74 were excluded for loss to follow-up or follow-up <3 months beyond the blanking period, yielding a final analytic cohort of 757 patients. The purpose of these criteria was to form a consistent study population and to limit confounding factors that could influence the dependability of the study findings. The study adhered to the principles outlined in the Declaration of Helsinki, and its protocol was approved by the Ethics Committee of Nanjing First Hospital. All patients provided written informed consent themselves. For patients over the age of 75, an additional dual-signature informed consent form was required, whereby a first-degree relative co-signed to enhance protection of patient rights. Therefore, a patient and one relative (first degree) had to sign the informed consent form. This measure was not a substitute for patient consent, and no legal guardians signed on a patient’s behalf.

## Cryoballoon ablation procedure

### Preoperative preparation

Before the procedure, a decapolar catheter was inserted into the coronary sinus, and a bipolar catheter was positioned at the right ventricular apex for pacing. Heparin was administered intravenously to maintain an activated clotting time (ACT) above 300 s. The procedure involved introducing a steerable sheath (FlexCath Advance, Medtronic, Minneapolis, MN, USA), a cryoballoon (Arctic Front Advance, Medtronic), and a circular mapping catheter (20-mm Achieve™, Medtronic) into the left atrium.

### Ablation protocol and procedural endpoints

The cryoballoon ablation strategy was standardized on the basis of the time-to-isolation (TTI) of the pulmonary vein potential (PVP). The protocol consisted of three scenarios: (1) For TTIs <30 s, an additional freezing duration of 120–150 s was applied after PVI was achieved; (2) for TTIs between 30–60 s, an initial 180-second freeze was followed by an additional 120-second application; and (3) for undetectable PVP or failure to achieve PVI within 60 s, the cryoballoon was repositioned, and the ablation sequence was repeated.

The procedure was terminated early if the balloon temperature reached ≤−55°C with a total ablation duration of <150 s to prevent complications. During right-sided PV isolation, continuous phrenic nerve stimulation was performed to monitor phrenic nerve function. If AF continued after PVI was completed, electrical cardioversion was used to reestablish sinus rhythm.

### Data collection

All potential predictive variables were collected before ablation. Age, sex, body mass index, type of atrial fibrillation (paroxysmal or persistent), duration of atrial fibrillation, and comorbid conditions such as hypertension, diabetes, and past stroke or TIA were included in the demographic and clinical characteristics. Risk stratification of all patients was performed via the CHA_2_DS_2_-VASc score. Blood samples were taken within 24 h prior to the procedure to obtain laboratory parameters, which included a multivariate Cox proportional hazards model complete blood count (white blood cells, red blood cells, hemoglobin) and B-type natriuretic peptide (BNP) levels. The cardiac evaluation included standard 12-lead ECG parameters recorded on the first postoperative day, including the heart rate, PR interval, and QT interval. Transesophageal echocardiography was performed before ablation to measure the left atrial diameter, left ventricular ejection fraction (LVEF), and left atrial appendage flow velocity (LAAFV) and to ensure that there were no intracardiac thrombi.

### Follow-up protocol and outcome assessment

A standardized follow-up protocol was implemented for all patients after ablation. AF recurrence was the primary outcome, characterized by any symptomatic or asymptomatic condition that lasted more than 30 seconds and was documented via ECG or Holter monitoring after a 3-month blanking period. Postprocedure follow-up visits were planned for 3, 6, and 12 months, followed by every 6 months thereafter. Patients were clinically assessed and underwent 24-hour Holter monitoring at each visit. All patients were required to undergo anticoagulation therapy for at least 3 months after the procedure using either vitamin K antagonists such as warfarin or direct oral anticoagulants such as rivaroxaban, edoxaban, or dabigatran. For patients unable to attend onsite follow-up visits, Holter monitoring reports from local hospitals were accepted, following our standardized protocol. To ensure data quality and completeness, all follow-up records were independently reviewed and verified by two experienced electrophysiologists.

### Statistical analyses and model construction and assessment

Statistical analyses were carried out via SPSS 24.0 and R 4.0.2, with statistical significance set at p<0.05. Variables with >30% missing values were excluded, whereas those with <30% missing values underwent multiple imputation via chained equations. The dataset was divided randomly into 70% for training and 30% for validation.

To compare baseline characteristics between the recurrence and nonrecurrence groups, categorical variables were analyzed via chi-square tests, whereas continuous variables were assessed via t tests or Mann‒Whitney U tests, as needed. Continuous variables are displayed as the mean ± standard deviation or median (interquartile range) on the basis of their distribution.

For predictor selection, we employed both LASSO regression and random forest algorithms on the 124 baseline variables in the training set. The glmnet package was used to conduct LASSO regression with 10-fold cross validation. To achieve a more parsimonious model, we deliberately increased the stringency of variable selection by setting the penalty parameter λ to 0.3 times the lambda.1 se value (lambda.1 se indicates the maximum λ that maintains the cross-validation error within one standard error of the minimum). Random forest analysis was used to rank variable importance on the basis of the mean decrease in impurities. Variables consistently identified by both methods were incorporated into a multivariate Cox proportional hazard model. The final model was visualized as a nomogram.

Model performance was evaluated via multiple metrics. Discrimination was assessed via time-dependent ROC curves and the C-index. Calibration was assessed via calibration plots, where perfect calibration was shown by a 45-degree line. Clinical utility was quantified via decision curve analysis. The model was internally validated in the validation cohort via identical metrics, and Kaplan‒Meier analysis was added to evaluate risk stratification.

To contextualize performance, we prespecified head-to-head benchmarking of the nomogram against APPLE, SUCCESS, PAT2C2H, HATCH, BASE-AF2, and CHA₂DS₂-VASc at 12 and 24 months. Scores were reconstructed per their original definitions as follows: APPLE (age >65 years, persistent AF, eGFR <60 mL/min/1.73 m^2^, LAD ≥43 mm, LVEF <50%; 1 point each); SUCCESS (APPLE +1 point per prior ablation); PAT2C2H (COPD, left-atrial enlargement, prior TIA/stroke, congestive heart failure, hypertension); HATCH (hypertension^1^, age ≥75^1^, TIA/stroke^2^, COPD^1^, heart failure^2^); BASE-AF2 (BMI >28 kg/m^2^, LA diameter >40 mm, current smoking, early recurrence after ablation, AF duration >6 years, nonparoxysmal AF; 1 point each); CHA₂DS₂-VASc (congestive heart failure, hypertension, age ≥75^2^, diabetes, stroke/TIA/TE^2^, vascular disease, age 65–74, female sex^1^). Time-dependent ROC analyses were performed at each horizon in both cohorts, with AUCs and 95% CIs estimated by nonparametric bootstrapping (B = 1000). Pairwise differences in discrimination were assessed via two-sided, paired DeLong tests for correlated ROC curves in the same participants, with Holm adjustment for multiplicity.

## Supplementary Information

Supplementary Information.

## Data Availability

Data from this study can be obtained from the corresponding author if requested reasonably, although they are not publicly accessible because they may contain sensitive participant details.

## Code availability

All custom R code used for statistical analyses in this study is provided as Supplementary Code File. The analyses include LASSO regression (glmnet package), random forest analysis (randomForest package), multivariate Cox proportional hazards modeling (survival package), time-dependent ROC curve analysis (timeROC package), calibration plots (rms package), decision curve analysis, and Kaplan-Meier survival analysis. All code was implemented in R version 4.0.2. SPSS 24.0 (IBM Corp., Armonk, NY, USA) was used for descriptive statistics and baseline characteristic comparisons. The R scripts include detailed comments to facilitate reproducibility and are available without restrictions for academic purposes. The complete code will be published as a supplementary document with this article to ensure version control and long-term accessibility.
