# Supplementary material for: Five-variable nomogram including PR interval and left atrial appendage flow velocity predicts atrial fibrillation recurrence after cryoballoon ablation
Source: Sci Rep. 2026 Jan 17;16:5644. doi: 10.1038/s41598-026-35653-9 (PMC12891680; doi:10.1038/s41598-026-35653-9)
Supplement: Supplementary file 1 — Supplementary Information. [file 41598_2026_35653_MOESM1_ESM.pdf]

Supplementary Table S1. Training cohort—discrimination at 12 and 24 months: c-statistic (AUC) with 95% confidence intervals and pairwise comparisons by DeLong's test. Interpretation.

Predicting scores	AUC at 12 months/24 months (c-statistic, 95% CI)	Nomo	CHA2DS2-VASc	APPLE	SUCCESS	PAT2C2H	HATCH
12 months							
Nomo	0.81 (0.77–0.86)						
CHA2DS2-VASc	0.53 (0.48–0.59)	<0.0001					
APPLE	0.70 (0.65–0.75)	0.0001	0.0001				
SUCCESS	0.70 (0.65–0.75)	<0.0001	0.0001	1.0000			
PAT2C2H	0.58 (0.52–0.64)	<0.0001	0.5411	0.0261	0.0295		
HATCH	0.53 (0.48–0.59)	<0.0001	1.0000	0.0003	0.0003	0.0120	
BASE_AF2	0.70 (0.65–0.75)	<0.0001	0.0152	1.0000	1.0000	0.1284	0.0055
24 months							
Nomo	0.83 (0.78–0.87)						
CHA2DS2-VASc	0.54 (0.47–0.60)	<0.0001					
APPLE	0.66 (0.60–0.72)	<0.0001	0.0006				
SUCCESS	0.65 (0.59–0.71)	<0.0001	0.0024	0.6917			
PAT2C2H	0.59 (0.53–0.65)	<0.0001	0.6917	0.1355	0.2280		
HATCH	0.54 (0.48–0.60)	<0.0001	1.0000	0.0015	0.0040	0.0123	
BASE_AF2	0.60 (0.54–0.66)	<0.0001	0.6917	0.2280	0.3732	1.0000	0.5122

The diagonal cells report the c-statistic (AUC) with 95% CI for each model at the specified horizon (12 or 24 months). Lower-triangle cells report two-sided, paired DeLong's P values comparing the row model with the column model using the same participants at that horizon (participants with ≥horizon follow-up or an event before the horizon); upper-triangle cells are intentionally blank.

Estimation and multiplicity control. The areas under the curve (AUCs) and 95% confidence intervals (CIs) were obtained via nonparametric bootstrapping (B = 1000). Within each cohort and horizon, P values from all pairwise comparisons among the K models (K = 7; 21 tests per horizon) were adjusted for multiplicity via the Holm method (familywise α = 0.05). Holm-adjusted P values are reported in Supplementary Tables S1–S2.

Abbreviations. AUC, area under the ROC curve; CI, confidence interval; Nomo, proposed nomogram.

Supplementary Table S2. Validation cohort—discrimination at 12 and 24 months: c-statistic (AUC) with 95% confidence intervals and pairwise comparisons by DeLong's test.

Interpretation. The diagonal cells report the c-statistic (AUC) with 95% CI for each model at the

Predicting scores	AUC at 12 months/24 months (c-statistic, 95% CI)	Nomo	CHA2DS2-VASc	APPLE	SUCCESS	PAT2C2H	HATCH
12 months							
Nomo	0.82 (0.75–0.89)						
CHA2DS2-VASc	0.52 (0.44–0.61)	<0.0001					
APPLE	0.64 (0.57–0.72)	0.0007	0.2365				
SUCCESS	0.64 (0.57–0.72)	0.0009	0.1817	1.0000			
PAT2C2H	0.57 (0.49–0.66)	0.0001	1.0000	1.0000	1.0000		
HATCH	0.51 (0.43–0.60)	<0.0001	1.0000	0.2823	0.2365	0.1038	
BASE_AF2	0.62 (0.54–0.70)	<0.0001	1.0000	1.0000	1.0000	1.0000	1.0000
24 months							
Nomo	0.80 (0.73–0.87)						
CHA2DS2-VASc	0.55 (0.45–0.63)	0.0001					
APPLE	0.65 (0.56–0.72)	0.0040	0.3976				
SUCCESS	0.65 (0.57–0.73)	0.0059	0.2945	1.0000			
PAT2C2H	0.56 (0.47–0.66)	0.0001	1.0000	0.8382	0.8375		
HATCH	0.57 (0.48–0.66)	0.0001	1.0000	0.8382	0.8375	1.0000	
BASE_AF2	0.67 (0.59–0.75)	0.0047	0.8375	1.0000	1.0000	0.8382	0.8382

specified horizon (12 or 24 months). Lower-triangle cells report two-sided, paired DeLong's P values comparing the row model with the column model using the same participants at that horizon (participants with ≥horizon follow-up or an event before the horizon); upper-triangle cells are intentionally blank.

Estimation and multiplicity control. The areas under the curve (AUCs) and 95% confidence intervals (CIs) were obtained via nonparametric bootstrapping (B = 1000). Within each cohort and horizon, P values from all pairwise comparisons among the K models (K = 7; 21 tests per horizon) were adjusted for multiplicity via the Holm method (familywise $\alpha = 0.05$). Holm-adjusted P values are reported in Supplementary Tables S1–S2.

Abbreviations. AUC, area under the ROC curve; CI, confidence interval; Nomo, proposed nomogram.

```
###############################################################
#################

# Title: Five-variable nomogram including PR interval and left atrial

#              appendage flow velocity predicts atrial fibrillation recurrence

#              after cryoballoon ablation

#

# Authors: Qiqiang Jie, Weichun Qian, Haibo Jia, Fengfu Zhang, Jianping Wang

#

# Institution: Department of Cardiology, Nanjing First Hospital,

#                       Nanjing Medical University, Nanjing, China

#

# Correspondence: Jianping Wang (jianpingwang_nexus@163.com)

#

# Description: This script contains all statistical analyses for predicting

#                       atrial fibrillation recurrence after cryoballoon ablation,

#                       including LASSO regression, random forest analysis, Cox

#                       proportional hazards modeling, nomogram construction,

#                       time-dependent ROC curves, calibration plots, decision curve

#                       analysis, Kaplan-Meier survival analysis, and benchmarking

#                       against established AF recurrence scores (APPLE, SUCCESS,

#                       PAT2C2H, HATCH, BASE-AF2, CHA2DS2-VASc) with paired DeLong

#                       tests and Holm adjustment for multiple comparisons.

#

# R Version: 4.0.2

# Date: December 2025

###############################################################
#################
```

```r
################################################################################
# SECTION 0: ENVIRONMENT SETUP
################################################################################


# Clear workspace
rm(list = ls())


# Load required packages
required_packages <- c(
  "glmnet",          # LASSO regression
  "randomForest",    # Random forest analysis
  "survival",        # Survival analysis
  "rms",             # Regression modeling strategies
  "timeROC",         # Time-dependent ROC curves
  "pROC",            # ROC analysis and DeLong tests
  "mice",            # Multiple imputation
  "dplyr",           # Data manipulation
  "ggplot2",         # Plotting
  "survminer",       # Survival plots
  "cowplot",         # Plot arrangements
  "janitor",         # Data cleaning
  "foreign",         # Data import
  "openxlsx"         # Excel file handling
)
```

```
# Install packages if not already installed
for (pkg in required_packages) {
  if (!require(pkg, character.only = TRUE)) {
    install.packages(pkg)
    library(pkg, character.only = TRUE)
  }
}

# Set working directory (MODIFY THIS PATH TO YOUR DIRECTORY)
setwd("C:/Users/jieqi/Desktop/AF CA 数据-chatgpt/AF-CA 结果分析")

cat("Environment setup complete.\n")
cat("R version:", R.version.string, "\n")
cat("All required packages loaded successfully.\n\n")

###################################################################
##################
# SECTION 1: LASSO REGRESSION ANALYSIS (TRAINING SET)
###################################################################
##################

cat("========================================\n")
cat("SECTION 1: LASSO REGRESSION ANALYSIS\n")
cat("========================================\n\n")

# Read training data for LASSO
```

```r
if (!file.exists("AFCA 训练集 LASSO2.csv")) {
  stop("File 'AFCA 训练集 LASSO2.csv' not found. Please check file path.")
}

dev <- tryCatch(
  read.csv("AFCA 训练集 LASSO2.csv",
           stringsAsFactors = FALSE,
           fileEncoding = "UTF-8-BOM",
           na.strings = c("", "NA")),
  error = function(e) stop("Error reading LASSO data file: ", e$message)
)

if (nrow(dev) == 0) {
  stop("LASSO data file is empty.")
}

# Clean column names and convert specified columns to factors
dev <- dev %>%
  clean_names() %>%
  mutate(across(99:124, as.factor))

# Multiple imputation for missing values
cat("Performing multiple imputation for missing values...\n")
imp <- mice(dev, m = 5, maxit = 50, method = 'pmm', seed = 500, printFlag = FALSE)
dev_imputed <- complete(imp, 1)

# Prepare predictor matrix
```

```
x <- dev_imputed[, c(99:124, 5:98)] %>%
  as.matrix() %>%
  apply(2, function(col) as.numeric(as.character(col)))
x[is.na(x)] <- 0

# Create survival object
dev_imputed$times <- as.numeric(as.character(dev_imputed$times))
y <- Surv(time = dev_imputed$times, event = dev_imputed$status)

# Fit LASSO regression with cross-validation
cat("Fitting LASSO regression model...\n")
fit <- glmnet(x, y, family = "cox")
cv.fit <- cv.glmnet(x, y, family = "cox", nfolds = 10)

# Plot LASSO coefficient paths
par(mar = c(5, 5, 4, 2) + 0.1)
plot(fit, xvar = "lambda", label = TRUE, main = "LASSO Coefficient Paths")

# Plot cross-validation curve
plot(cv.fit, main = "LASSO Cross-Validation")
abline(v = log(c(cv.fit$lambda.min, cv.fit$lambda.1se)), lty = 2)

# Use higher lambda for more parsimonious model
lambda_high <- cv.fit$lambda.1se * 0.3
Coefficients <- coef(fit, s = lambda_high)
Active.Index <- which(Coefficients != 0)
Active.Coefficients <- Coefficients[Active.Index]
```

```r
cat("\nLASSO-selected variables (lambda =", round(lambda_high, 4), "):\n")
cat("Number of selected variables:", length(Active.Index), "\n")
print(data.frame(
  Variable = row.names(Coefficients)[Active.Index],
  Coefficient = as.numeric(Active.Coefficients)
))

lasso_variables <- row.names(Coefficients)[Active.Index]

###############################################################
#################
# SECTION 2: RANDOM FOREST ANALYSIS (TRAINING SET)
###############################################################
#################

cat("\n========================================\n")
cat("SECTION 2: RANDOM FOREST ANALYSIS\n")
cat("========================================\n\n")

# Read training data for random forest
dev_rf <- read.csv("AFCA 训练集随机森林.csv", fileEncoding = "GBK")

if (nrow(dev_rf) == 0) {
  stop("Random forest data file is empty.")
}
```

```
# Multiple imputation
cat("Performing multiple imputation for random forest data...\n")
imp_rf <- mice(dev_rf, m = 5, maxit = 50, method = 'pmm', seed = 500, printFlag =
FALSE)
dev_rf_imputed <- complete(imp_rf, 1)

# Prepare features
x_rf <- dev_rf_imputed[, c(96:121, 5:95)] %>%
  as.matrix() %>%
  apply(2, function(col) as.numeric(as.character(col)))
x_rf[is.na(x_rf)] <- 0

# Prepare target variable
y_rf <- as.factor(dev_rf_imputed$status)

# Run random forest model
cat("Running random forest model...\n")
set.seed(123)
rf_model <- randomForest(
  x_rf, y_rf,
  importance = TRUE,
  ntree = 500,
  mtry = sqrt(ncol(x_rf))
)

cat("\nRandom Forest Results:\n")
print(rf_model$confusion)
```

```r
# Extract top 20 important variables
importance_data <- data.frame(
  Variable = row.names(rf_model$importance),
  Importance = rf_model$importance[, "MeanDecreaseGini"]
) %>%
  arrange(desc(Importance)) %>%
  slice_head(n = 20)

cat("\nTop 20 important variables:\n")
print(importance_data)

rf_variables <- importance_data$Variable

# Plot variable importance
importance_plot <- ggplot(importance_data,
                          aes(x = reorder(Variable, Importance), y = Importance)) +
  geom_bar(stat = 'identity', fill = 'red') +
  coord_flip() +
  labs(
    title = "Top 20 Variable Importance - Random Forest",
    x = "Variables",
    y = "Mean Decrease Gini"
  ) +
  theme_minimal() +
  theme(
    plot.title = element_text(size = 14, face = "bold"),
```

```
    axis.text = element_text(size = 10),
    axis.title = element_text(size = 12)
  )

print(importance_plot)

# Save plot
ggsave("random_forest_top20_importance.png", importance_plot,
       width = 12, height = 10, dpi = 300)

###################################################################
##################
# SECTION 3: VARIABLE INTERSECTION AND COX REGRESSION
###################################################################
##################

cat("\n==================================================\n")
cat("SECTION 3: FINAL MODEL CONSTRUCTION\n")
cat("==================================================\n\n")

# Identify overlapping variables from LASSO and Random Forest
# Based on manuscript: Female, PeAF, PR_interval, LA, LAAFV
cat("Final selected variables for nomogram:\n")
cat("1. Female (sex)\n")
cat("2. PeAF (persistent AF)\n")
cat("3. PR_interval (PR interval)\n")
cat("4. LA (left atrial diameter)\n")
```

```r
cat("5. LAAFV (left atrial appendage flow velocity)\n\n")

# Read training data for nomogram construction
dev_nomo <- read.csv("AFCA 训练集列线图.csv")
dev_nomo <- na.omit(dev_nomo)

# Convert factors
dev_nomo$PeAF <- factor(dev_nomo$PeAF, levels = c(0, 1), labels = c("No", "Yes"))
dev_nomo$Female <- factor(dev_nomo$Female, levels = c(0, 1), labels = c("No", "Yes"))

# Set datadist
ddist <- datadist(dev_nomo)
options(datadist = 'ddist')

# Set time units
units(dev_nomo$times) <- "Month"

# Build Cox proportional hazards model
cat("Building Cox proportional hazards model...\n")
fcox <- cph(Surv(times, status) ~ Female + PeAF + PR_interval + LA + LAAFV,
            surv = TRUE, x = TRUE, y = TRUE, data = dev_nomo)

cat("\nCox Model Summary:\n")
print(fcox)

# Create survival function
surv <- Survival(fcox)
```

```
# Construct nomogram
cat("\nConstructing nomogram...\n")
nom <- nomogram(
  fcox,
  fun = list(
    function(x) surv(12, x),
    function(x) surv(24, x)
  ),
  funlabel = c("1-year Survival Probability", "2-years Survival Probability"),
  lp = FALSE
)

# Plot nomogram
plot(nom, lwd = 2, col.grid = gray(0.9), cex.axis = 0.8,
     total.points.at = seq(0, 400, by = 50))

cat("\nNomogram construction complete.\n")

# Calculate nomogram scores for training set
library(nomogramEx)
nomogramEx(nomo = nom, np = 2, digit = 5)

# Calculate points for each predictor
dev_nomo$female_point <- ifelse(dev_nomo$Female == "Yes", 53.82904, 0)
dev_nomo$peaf_point <- ifelse(dev_nomo$PeAF == "Yes", 72.48439, 0)
dev_nomo$pr_point <- 1.09366 * dev_nomo$PR_interval - 131.23966
```

```
dev_nomo$la_point <- 1.9802 * dev_nomo$LA - 59.40593
dev_nomo$laafv_point <- -1.11111 * dev_nomo$LAAFV + 122.22222

# Calculate total points
dev_nomo$total_points <- dev_nomo$female_point + dev_nomo$peaf_point +
  dev_nomo$pr_point + dev_nomo$la_point + dev_nomo$laafv_point

# Calculate survival probabilities
dev_nomo$prob_1year <- -0.00005 * dev_nomo$total_points^2 +
  0.00846 * dev_nomo$total_points + 0.47194
dev_nomo$prob_2year <- -0.00004 * dev_nomo$total_points^2 +
  0.00289 * dev_nomo$total_points + 0.83709

# Save results
write.csv(dev_nomo, "AFCA 训练集列线图计算后患者总点数.csv", row.names = FALSE)

##################################################################
##################
# SECTION 4: TIME-DEPENDENT ROC CURVES (TRAINING SET)
##################################################################
##################

cat("\n========================================\n")
cat("SECTION 4: TIME-DEPENDENT ROC - TRAINING SET\n")
cat("========================================\n\n")

# Read training data for ROC
```

```r
dev_roc_train <- read.csv("AFCA 训练集 ROC.csv")

# Convert variables to numeric
dev_roc_train$Female <- as.numeric(as.character(dev_roc_train$Female))
dev_roc_train$PeAF <- as.numeric(as.character(dev_roc_train$PeAF))
dev_roc_train$PR_interval <- as.numeric(as.character(dev_roc_train$PR_interval))
dev_roc_train$LA <- as.numeric(as.character(dev_roc_train$LA))
dev_roc_train$LAAFV <- as.numeric(as.character(dev_roc_train$LAAFV))

# Set datadist
ddist <- datadist(dev_roc_train)
options(datadist = 'ddist')

# Filter complete cases
complete_cases <- complete.cases(dev_roc_train$follow, dev_roc_train$status,
                                  dev_roc_train$Female, dev_roc_train$PeAF,
                                  dev_roc_train$PR_interval, dev_roc_train$LA,
                                  dev_roc_train$LAAFV)
model_data_train <- dev_roc_train[complete_cases, ]

# Build Cox model
fmla1 <- as.formula(Surv(follow, status) ~ Female + PeAF + PR_interval + LA + LAAFV)
cox_train <- coxph(fmla1, data = model_data_train)

cat("Cox Model for Training Set:\n")
print(summary(cox_train))
```

```r
# Calculate risk scores
risk_scores_train <- predict(cox_train, type = "risk")

# Calculate time-dependent ROC
cat("\nCalculating time-dependent ROC curves...\n")
roc_train <- timeROC(
  T = model_data_train$follow,
  delta = model_data_train$status,
  marker = risk_scores_train,
  cause = 1,
  times = c(12, 24),
  iid = TRUE
)

# Bootstrap for confidence intervals
cat("Performing bootstrap for confidence intervals (n=1000)...\n")
set.seed(123)
n_boot <- 1000
auc_boot_12_train <- numeric(n_boot)
auc_boot_24_train <- numeric(n_boot)

for (i in 1:n_boot) {
  if (i %% 100 == 0) cat("Bootstrap iteration:", i, "/", n_boot, "\n")
  boot_idx <- sample(1:nrow(model_data_train), replace = TRUE)
  boot_data <- model_data_train[boot_idx, ]
  boot_cox <- coxph(fmla1, data = boot_data)
  boot_risk_scores <- predict(boot_cox, type = "risk")
```

```
  boot_roc <- timeROC(
    T = boot_data$follow,
    delta = boot_data$status,
    marker = boot_risk_scores,
    cause = 1,
    times = c(12, 24),
    iid = FALSE
  )
  auc_boot_12_train[i] <- boot_roc$AUC[1]
  auc_boot_24_train[i] <- boot_roc$AUC[2]
}

# Calculate 95% CI
ci_boot_12_train <- quantile(auc_boot_12_train, probs = c(0.025, 0.975))
ci_boot_24_train <- quantile(auc_boot_24_train, probs = c(0.025, 0.975))

cat("\nTraining Set ROC Results:\n")
cat("12-month AUC:", round(roc_train$AUC[1], 3),
    "95% CI: [", round(ci_boot_12_train[1], 3), "-", round(ci_boot_12_train[2], 3), "]\n")
cat("24-month AUC:", round(roc_train$AUC[2], 3),
    "95% CI: [", round(ci_boot_24_train[1], 3), "-", round(ci_boot_24_train[2], 3), "]\n")

# Plot ROC curves
par(mar = c(5, 5, 4, 2))
plot(roc_train, time = 12, col = "red", lwd = 4, title = "", lty = 1)
plot(roc_train, time = 24, add = TRUE, col = "blue", lwd = 4, lty = 1)
abline(a = 0, b = 1, lty = 2)
```

```
legend_text_train <- c(
  sprintf("12 months (AUC = %.2f, 95%% CI = [%.2f, %.2f])",
          roc_train$AUC[1], ci_boot_12_train[1], ci_boot_12_train[2]),
  sprintf("24 months (AUC = %.2f, 95%% CI = [%.2f, %.2f])",
          roc_train$AUC[2], ci_boot_24_train[1], ci_boot_24_train[2])
)

legend(x = 0.3, y = 0.05, legend = legend_text_train,
       col = c("red", "blue"), lwd = 2, lty = 1, bty = "n", cex = 0.9)

title(xlab = "1-Specificity", ylab = "Sensitivity")

###################################################################
##################
# SECTION 5: TIME-DEPENDENT ROC CURVES (VALIDATION SET)
###################################################################
##################

cat("\n========================================\n")
cat("SECTION 5: TIME-DEPENDENT ROC - VALIDATION SET\n")
cat("========================================\n\n")

# Read validation data
dev_roc_val <- read.csv("AFCA 验证集列线图修改后.csv")

# Convert variables to numeric
```

```
dev_roc_val$Female <- as.numeric(as.character(dev_roc_val$Female))
dev_roc_val$PeAF <- as.numeric(as.character(dev_roc_val$PeAF))
dev_roc_val$PR_interval <- as.numeric(as.character(dev_roc_val$PR_interval))
dev_roc_val$LA <- as.numeric(as.character(dev_roc_val$LA))
dev_roc_val$LAAFV <- as.numeric(as.character(dev_roc_val$LAAFV))

# Filter complete cases
complete_cases_val <- complete.cases(dev_roc_val$follow, dev_roc_val$status,
                                     dev_roc_val$Female, dev_roc_val$PeAF,
                                     dev_roc_val$PR_interval, dev_roc_val$LA,
                                     dev_roc_val$LAAFV)
model_data_val <- dev_roc_val[complete_cases_val, ]

# Build Cox model
cox_val <- coxph(fmla1, data = model_data_val)

cat("Cox Model for Validation Set:\n")
print(summary(cox_val))

# Calculate risk scores
risk_scores_val <- predict(cox_val, type = "risk")

# Calculate time-dependent ROC
roc_val <- timeROC(
  T = model_data_val$follow,
  delta = model_data_val$status,
  marker = risk_scores_val,
```

```
    cause = 1,
    times = c(12, 24),
    iid = TRUE
)

# Bootstrap for confidence intervals
cat("\nPerforming bootstrap for validation set (n=1000)...\n")
set.seed(123)
auc_boot_12_val <- numeric(n_boot)
auc_boot_24_val <- numeric(n_boot)

for (i in 1:n_boot) {
    if (i %% 100 == 0) cat("Bootstrap iteration:", i, "/", n_boot, "\n")
    boot_idx <- sample(1:nrow(model_data_val), replace = TRUE)
    boot_data <- model_data_val[boot_idx, ]
    boot_cox <- coxph(fmla1, data = boot_data)
    boot_risk_scores <- predict(boot_cox, type = "risk")
    boot_roc <- timeROC(
        T = boot_data$follow,
        delta = boot_data$status,
        marker = boot_risk_scores,
        cause = 1,
        times = c(12, 24),
        iid = FALSE
    )
    auc_boot_12_val[i] <- boot_roc$AUC[1]
    auc_boot_24_val[i] <- boot_roc$AUC[2]
```

```r
}

# Calculate 95% CI
ci_boot_12_val <- quantile(auc_boot_12_val, probs = c(0.025, 0.975))
ci_boot_24_val <- quantile(auc_boot_24_val, probs = c(0.025, 0.975))

cat("\nValidation Set ROC Results:\n")
cat("12-month AUC:", round(roc_val$AUC[1], 3),
    "95% CI: [", round(ci_boot_12_val[1], 3), "-", round(ci_boot_12_val[2], 3), "]\n")
cat("24-month AUC:", round(roc_val$AUC[2], 3),
    "95% CI: [", round(ci_boot_24_val[1], 3), "-", round(ci_boot_24_val[2], 3), "]\n")

# Plot ROC curves
par(mar = c(5, 5, 4, 2))
plot(roc_val, time = 12, col = "red", lwd = 4, title = "", lty = 1)
plot(roc_val, time = 24, add = TRUE, col = "blue", lwd = 4, lty = 1)
abline(a = 0, b = 1, lty = 2)

legend_text_val <- c(
  sprintf("12 months (AUC = %.2f, 95%% CI = [%.2f, %.2f])",
          roc_val$AUC[1], ci_boot_12_val[1], ci_boot_12_val[2]),
  sprintf("24 months (AUC = %.2f, 95%% CI = [%.2f, %.2f])",
          roc_val$AUC[2], ci_boot_24_val[1], ci_boot_24_val[2])
)

legend(x = 0.3, y = 0.05, legend = legend_text_val,
       col = c("red", "blue"), lwd = 2, lty = 1, bty = "n", cex = 0.9)
```

```r
title(xlab = "1-Specificity", ylab = "Sensitivity")

################################################################################
# SECTION 6: CALIBRATION CURVES (TRAINING SET)
################################################################################

cat("\n========================================\n")
cat("SECTION 6: CALIBRATION CURVES - TRAINING SET\n")
cat("========================================\n\n")

# Read training data for calibration
dev_cal_train <- read.csv("AFCA 训练集列线图 2 修改后.csv")

# Convert factors
dev_cal_train$PeAF <- factor(dev_cal_train$PeAF, levels = c(0, 1),
                             labels = c("No", "Yes"))
dev_cal_train$Female <- factor(dev_cal_train$Female, levels = c(0, 1),
                               labels = c("No", "Yes"))

# Set datadist
ddist <- datadist(dev_cal_train)
options(datadist = 'ddist')
units(dev_cal_train$follow) <- "Month"
```

```
# Build Cox models for calibration
cat("Building Cox models for calibration (training set)...\n")
fcox_cal_train_12 <- cph(Surv(follow, status) ~ Female + PeAF + PR_interval + LA + LAAFV,
                          surv = TRUE, x = TRUE, y = TRUE, time.inc = 12,
                          data = dev_cal_train)
cal_train_12 <- calibrate(fcox_cal_train_12, cmethod = "KM", method = "boot",
                           u = 12, m = 60, B = 1000)

fcox_cal_train_24 <- cph(Surv(follow, status) ~ Female + PeAF + PR_interval + LA + LAAFV,
                          surv = TRUE, x = TRUE, y = TRUE, time.inc = 24,
                          data = dev_cal_train)
cal_train_24 <- calibrate(fcox_cal_train_24, cmethod = "KM", method = "boot",
                           u = 24, m = 60, B = 500)

# Plot calibration curves
par(mar = c(5, 5, 4, 2))
plot(0, 0, type = "n", xlim = c(0, 1), ylim = c(0, 1),
     xlab = "Predicted Survival", ylab = "Actual Survival",
     main = "Calibration Curves - Training Set",
     cex.lab = 1.2, cex.axis = 1.1, cex.main = 1.3)

grid(nx = NULL, ny = NULL, lty = 2, col = "gray90")
abline(0, 1, col = "gray50", lty = 2)

col1 <- "red"
```

```r
col2 <- "blue"

plot(cal_train_12, add = TRUE, col = col1, lwd = 2, errbar.col = col1)
plot(cal_train_24, add = TRUE, col = col2, lwd = 2, errbar.col = col2)

legend("topleft",
       legend = c("1-year", "2-year", "Ideal"),
       col = c(col1, col2, "gray50"),
       lty = c(1, 1, 2),
       lwd = c(2, 2, 1),
       bty = "n",
       cex = 1.1,
       inset = 0.02)

text(0.2, 0.1, "Lines include 95% confidence intervals",
     cex = 0.8, col = "gray40")

cat("\nCalibration curves for training set completed.\n")

###############################################################################
# SECTION 7: CALIBRATION CURVES (VALIDATION SET)
###############################################################################

cat("\n========================================\n")
cat("SECTION 7: CALIBRATION CURVES - VALIDATION SET\n")
```

```r
cat("=============================================\n\n")

# Data already loaded (dev_roc_val)
dev_cal_val <- dev_roc_val

# Convert factors
dev_cal_val$PeAF <- factor(dev_cal_val$PeAF, levels = c(0, 1),
                           labels = c("No", "Yes"))
dev_cal_val$Female <- factor(dev_cal_val$Female, levels = c(0, 1),
                             labels = c("No", "Yes"))

# Set datadist
ddist <- datadist(dev_cal_val)
options(datadist = 'ddist')
units(dev_cal_val$follow) <- "Month"

# Build Cox models for calibration
cat("Building Cox models for calibration (validation set)...\n")
fcox_cal_val_12 <- cph(Surv(follow, status) ~ Female + PeAF + PR_interval + LA +
LAAFV,
                       surv = TRUE, x = TRUE, y = TRUE, time.inc = 12,
                       data = dev_cal_val)
cal_val_12 <- calibrate(fcox_cal_val_12, cmethod = "KM", method = "boot",
                        u = 12, m = 60, B = 1000)

fcox_cal_val_24 <- cph(Surv(follow, status) ~ Female + PeAF + PR_interval + LA +
LAAFV,
```

```
                                   surv = TRUE, x = TRUE, y = TRUE, time.inc = 24,
                                   data = dev_cal_val)
cal_val_24 <- calibrate(fcox_cal_val_24, cmethod = "KM", method = "boot",
                                    u = 24, m = 60, B = 500)

# Plot calibration curves
par(mar = c(5, 5, 4, 2))
plot(0, 0, type = "n", xlim = c(0, 1), ylim = c(0, 1),
       xlab = "Predicted Survival", ylab = "Actual Survival",
       main = "Calibration Curves - Validation Set",
       cex.lab = 1.2, cex.axis = 1.1, cex.main = 1.3)

grid(nx = NULL, ny = NULL, lty = 2, col = "gray90")
abline(0, 1, col = "gray50", lty = 2)

plot(cal_val_12, add = TRUE, col = col1, lwd = 2, errbar.col = col1)
plot(cal_val_24, add = TRUE, col = col2, lwd = 2, errbar.col = col2)

legend("topleft",
          legend = c("1-year", "2-year", "Ideal"),
          col = c(col1, col2, "gray50"),
          lty = c(1, 1, 2),
          lwd = c(2, 2, 1),
          bty = "n",
          cex = 1.1,
          inset = 0.02)
```

```
text(0.2, 0.1, "Lines include 95% confidence intervals",
     cex = 0.8, col = "gray40")

cat("\nCalibration curves for validation set completed.\n")

#################################################################################
# SECTION 8: DECISION CURVE ANALYSIS (TRAINING SET)
#################################################################################

cat("\n========================================\n")
cat("SECTION 8: DECISION CURVE ANALYSIS - TRAINING SET\n")
cat("========================================\n\n")

# NOTE: This section requires the stdca.R file
# Make sure stdca.R is in your working directory
if (!file.exists("stdca.R")) {
  cat("WARNING: stdca.R file not found. Skipping DCA analysis.\n")
  cat("Please download stdca.R from: https://www.mskcc.org/departments/epidemiology-biostatistics/biostatistics/decision-curve-analysis\n")
} else {
  source("stdca.R")

  # Read DCA training data
  data_dca_train <- read.csv("AFCA 训练集列线图 2 修改后.csv")
  data_dca_train <- na.omit(data_dca_train)
```

```
# Convert factors
data_dca_train$PeAF <- factor(data_dca_train$PeAF, levels = c(0, 1),
                              labels = c("No", "Yes"))
data_dca_train$Female <- factor(data_dca_train$Female, levels = c(0, 1),
                                labels = c("No", "Yes"))

# Ensure numeric variables
data_dca_train$PR_interval <- as.numeric(data_dca_train$PR_interval)
data_dca_train$LA <- as.numeric(data_dca_train$LA)
data_dca_train$LAAFV <- as.numeric(data_dca_train$LAAFV)

# Create survival object
Srv <- Surv(data_dca_train$follow, data_dca_train$status)

# Build Cox model
coxmod_dca <- coxph(Srv ~ Female + PeAF + PR_interval + LA + LAAFV,
                    data = data_dca_train)

# Calculate survival probabilities
surv_fit_dca <- survfit(coxmod_dca, newdata = data_dca_train)

# 1-year survival probability (convert to event probability)
data_dca_train$one.year.Survival.Probabilitynew <-
  c(1 - (summary(survfit(coxmod_dca, newdata = data_dca_train), times = 12)$surv))

# 2-year survival probability (convert to event probability)
```

```
data_dca_train$two.years.Survival.Probabilitynew <-
  c(1 - (summary(survfit(coxmod_dca, newdata = data_dca_train), times = 24)$surv))

# Save DCA data
write.csv(data_dca_train, "训练集 DCA.csv", row.names = FALSE)

cat("Running DCA for 1-year prediction...\n")
stdca(data = data_dca_train,
      outcome = "status",
      ttoutcome = "follow",
      timepoint = 12,
      predictors = "one.year.Survival.Probabilitynew",
      xstop = 0.9,
      smooth = TRUE)

cat("Running DCA for 2-year prediction...\n")
stdca(data = data_dca_train,
      outcome = "status",
      ttoutcome = "follow",
      timepoint = 24,
      predictors = "two.years.Survival.Probabilitynew",
      xstart = 0.1,
      xstop = 1.0,
      smooth = FALSE,
      probability = TRUE,
      harm = 0,
      intervention = FALSE)
```

```
    cat("\nDCA for training set completed.\n")
}

#########################################################################
#################
# SECTION 9: DECISION CURVE ANALYSIS (VALIDATION SET)
#########################################################################
#################

cat("\n===========================================\n")
cat("SECTION 9: DECISION CURVE ANALYSIS - VALIDATION SET\n")
cat("===========================================\n\n")

if (!file.exists("stdca.R")) {
    cat("WARNING: stdca.R file not found. Skipping validation DCA.\n")
} else {
    # Read validation data for DCA
    data_dca_val <- read.csv("AFCA 验证集列线图修改后.csv")
    data_dca_val <- na.omit(data_dca_val)

    # Convert factors
    data_dca_val$PeAF <- factor(data_dca_val$PeAF, levels = c(0, 1),
                                            labels = c("No", "Yes"))
    data_dca_val$Female <- factor(data_dca_val$Female, levels = c(0, 1),
                                              labels = c("No", "Yes"))
```

```
# Ensure numeric variables
data_dca_val$PR_interval <- as.numeric(data_dca_val$PR_interval)
data_dca_val$LA <- as.numeric(data_dca_val$LA)
data_dca_val$LAAFV <- as.numeric(data_dca_val$LAAFV)

# Create survival object
Srv_val <- Surv(data_dca_val$follow, data_dca_val$status)

# Build Cox model
coxmod_dca_val <- coxph(Srv_val ~ Female + PeAF + PR_interval + LA + LAAFV,
                        data = data_dca_val)

# Calculate survival probabilities
data_dca_val$one.year.Survival.Probabilitynew <-
  c(1 - (summary(survfit(coxmod_dca_val, newdata = data_dca_val), times =
12)$surv))

data_dca_val$two.years.Survival.Probabilitynew <-
  c(1 - (summary(survfit(coxmod_dca_val, newdata = data_dca_val), times =
24)$surv))

# Save DCA data
write.csv(data_dca_val, "验证集 DCA.csv", row.names = FALSE)

cat("Running DCA for 1-year prediction (validation)...\n")
stdca(data = data_dca_val,
      outcome = "status",
```

```
            ttoutcome = "follow",
            timepoint = 12,
            predictors = "one.year.Survival.Probabilitynew",
            xstop = 0.9,
            smooth = TRUE)

   cat("Running DCA for 2-year prediction (validation)...\n")
   stdca(data = data_dca_val,
            outcome = "status",
            ttoutcome = "follow",
            timepoint = 24,
            predictors = "two.years.Survival.Probabilitynew",
            xstart = 0.1,
            xstop = 1.0,
            smooth = FALSE,
            probability = TRUE,
            harm = 0,
            intervention = FALSE)

   cat("\nDCA for validation set completed.\n")
}

#################################################################################
# SECTION 10: KAPLAN-MEIER SURVIVAL ANALYSIS (TRAINING SET)
#################################################################################
```

```
cat("\n==========================================\n")
cat("SECTION 10: KAPLAN-MEIER ANALYSIS - TRAINING SET\n")
cat("==========================================\n\n")

# Read training data with calculated total points
data_km_train <- read.csv("AFCA 训练集列线图计算后患者总点数.csv")

# Calculate tertiles for risk stratification
quartiles_train <- quantile(data_km_train$total_points, probs = c(1/3, 2/3))

cat("Risk stratification cutoff values (tertiles):\n")
cat("Low vs Intermediate:", round(quartiles_train[1], 2), "\n")
cat("Intermediate vs High:", round(quartiles_train[2], 2), "\n\n")

# Assign risk groups
data_km_train <- data_km_train %>%
  mutate(risk_group = case_when(
    total_points <= quartiles_train[1] ~ "Low Risk",
    total_points <= quartiles_train[2] ~ "Intermediate Risk",
    TRUE ~ "High Risk"
  ))

data_km_train$risk_group <- factor(data_km_train$risk_group,
                                   levels = c("Low Risk", "Intermediate Risk", "High
Risk"))
```

```
cat("Risk group distribution:\n")
print(table(data_km_train$risk_group))
cat("\n")

# Fit Kaplan-Meier survival curves
fit_train <- survfit(Surv(follow, status) ~ risk_group, data = data_km_train)

# Log-rank test
logrank_test_train <- survdiff(Surv(follow, status) ~ risk_group,
                               data = data_km_train)
p_value_train <- 1 - pchisq(logrank_test_train$chisq,
                            length(logrank_test_train$n) - 1)

cat("Log-Rank Test Results (Training Set):\n")
print(logrank_test_train)
cat("\nP-value:", format.pval(p_value_train, digits = 3), "\n\n")

# Create p-value text
p_text_train <- if(p_value_train < 0.001) {
  "P < 0.001"
} else {
  sprintf("P = %.3f", p_value_train)
}

# Plot Kaplan-Meier curves
km_plot_train <- ggsurvplot(
  fit_train,
```

```
  data = data_km_train,
  risk.table = TRUE,
  conf.int = TRUE,
  linetype = "strata",
  surv.median.line = "hv",
  ggtheme = theme_classic(),
  palette = c("#2E9FDF", "#E7B800", "#FC4E07"),
  risk.table.y.text.col = TRUE,
  risk.table.y.text = FALSE,
  risk.table.height = 0.3,
  xlim = c(0, 36),
  break.time.by = 12,
  risk.table.title = "Number at risk",
  risk.table.theme = theme_minimal(),
  tables.theme = theme_classic(),
  font.main = c(14, "bold", "black"),
  font.x = c(14, "bold", "black"),
  font.y = c(14, "bold", "black"),
  font.tickslab = c(14),
  risk.table.fontsize = 4
)

# Customize plot
km_plot_train$plot <- km_plot_train$plot +
  scale_x_continuous(breaks = c(0, 12, 24, 36),
                     labels = c("0", "12", "24", "36"),
                     expand = c(0.05, 0)) +
```

```
  scale_y_continuous(expand = c(0, 0)) +
  labs(x = "Time (months)", title = "Training Set") +
  annotate("text", x = 5, y = 0.1, label = p_text_train,
           size = 4.5, fontface = "bold") +
  theme(
    axis.line = element_line(size = 1.5, colour = "black"),
    axis.ticks = element_line(size = 1.2),
    axis.text = element_text(size = 14),
    axis.title.x = element_text(size = 14, face = "bold", colour = "black")
  )

km_plot_train$table <- km_plot_train$table +
  scale_x_continuous(breaks = c(0, 12, 24, 36),
                     labels = c("0", "12", "24", "36"),
                     expand = c(0.05, 0)) +
  theme(
    axis.line = element_line(size = 1.5, colour = "black"),
    axis.ticks = element_line(size = 1.2),
    axis.text = element_text(size = 14),
    axis.title.x = element_text(size = 14, face = "bold", colour = "black"),
    plot.title = element_text(size = 14, face = "bold", colour = "black")
  )

# Align and print
aligned_plots_train <- align_plots(km_plot_train$plot, km_plot_train$table,
                                   align = "v", axis = "lr")
final_plot_train <- plot_grid(aligned_plots_train[[1]], aligned_plots_train[[2]],
```

```
                                        ncol = 1, rel_heights = c(3, 1))
print(final_plot_train)

cat("\nKaplan-Meier analysis for training set completed.\n")

################################################################################
# SECTION 11: KAPLAN-MEIER SURVIVAL ANALYSIS (VALIDATION SET)
################################################################################

cat("\n========================================\n")
cat("SECTION 11: KAPLAN-MEIER ANALYSIS - VALIDATION SET\n")
cat("========================================\n\n")

# Read validation data
data_km_val <- read.csv("AFCA 验证集列线图修改后根据训练集参数计算得到总点数2.csv")

# Use fixed cutoff values from training set
cat("Using training set cutoff values:\n")
cat("Low vs Intermediate:", round(quartiles_train[1], 4), "\n")
cat("Intermediate vs High:", round(quartiles_train[2], 4), "\n\n")

# Assign risk groups using training set cutoffs
data_km_val <- data_km_val %>%
  mutate(risk_group = case_when(
    total_points <= 159.7844 ~ "Low Risk",
```

```
        total_points <= 216.9318 ~ "Intermediate Risk",
        TRUE ~ "High Risk"
    ))

data_km_val$risk_group <- factor(data_km_val$risk_group,
                                 levels = c("Low Risk", "Intermediate Risk", "High
Risk"))

cat("Risk group distribution (validation set):\n")
print(table(data_km_val$risk_group))
cat("\n")

# Fit Kaplan-Meier survival curves
fit_val <- survfit(Surv(follow, status) ~ risk_group, data = data_km_val)

# Log-rank test
logrank_test_val <- survdiff(Surv(follow, status) ~ risk_group,
                             data = data_km_val)
p_value_val <- 1 - pchisq(logrank_test_val$chisq,
                          length(logrank_test_val$n) - 1)

cat("Log-Rank Test Results (Validation Set):\n")
print(logrank_test_val)
cat("\nP-value:", format.pval(p_value_val, digits = 3), "\n\n")

# Create p-value text
p_text_val <- if(p_value_val < 0.001) {
```

```
    "P < 0.001"
} else {
    sprintf("P = %.3f", p_value_val)
}

# Plot Kaplan-Meier curves
km_plot_val <- ggsurvplot(
    fit_val,
    data = data_km_val,
    risk.table = TRUE,
    conf.int = TRUE,
    linetype = "strata",
    surv.median.line = "hv",
    ggtheme = theme_classic(),
    palette = c("#2E9FDF", "#E7B800", "#FC4E07"),
    risk.table.y.text.col = TRUE,
    risk.table.y.text = FALSE,
    risk.table.height = 0.3,
    xlim = c(0, 36),
    break.time.by = 12,
    risk.table.title = "Number at risk",
    risk.table.theme = theme_minimal(),
    tables.theme = theme_classic(),
    font.main = c(14, "bold", "black"),
    font.x = c(14, "bold", "black"),
    font.y = c(14, "bold", "black"),
    font.tickslab = c(14),
```

```
  risk.table.fontsize = 4
)

# Customize plot
km_plot_val$plot <- km_plot_val$plot +
  scale_x_continuous(breaks = c(0, 12, 24, 36),
                     labels = c("0", "12", "24", "36"),
                     expand = c(0.05, 0)) +
  scale_y_continuous(expand = c(0, 0)) +
  labs(x = "Time (months)", title = "Validation Set") +
  annotate("text", x = 5, y = 0.1, label = p_text_val,
           size = 4.5, fontface = "bold") +
  theme(
    axis.line = element_line(size = 1.5, colour = "black"),
    axis.ticks = element_line(size = 1.2),
    axis.text = element_text(size = 14),
    axis.title.x = element_text(size = 14, face = "bold", colour = "black")
  )

km_plot_val$table <- km_plot_val$table +
  scale_x_continuous(breaks = c(0, 12, 24, 36),
                     labels = c("0", "12", "24", "36"),
                     expand = c(0.05, 0)) +
  theme(
    axis.line = element_line(size = 1.5, colour = "black"),
    axis.ticks = element_line(size = 1.2),
    axis.text = element_text(size = 14),
```

```r
      axis.title.x = element_text(size = 14, face = "bold", colour = "black"),
      plot.title = element_text(size = 14, face = "bold", colour = "black")
    )

# Align and print
aligned_plots_val <- align_plots(km_plot_val$plot, km_plot_val$table,
                                  align = "v", axis = "lr")
final_plot_val <- plot_grid(aligned_plots_val[[1]], aligned_plots_val[[2]],
                             ncol = 1, rel_heights = c(3, 1))
print(final_plot_val)

cat("\nKaplan-Meier analysis for validation set completed.\n")

###################################################################
##################
# SECTION 12: COMPARISON WITH OTHER SCORES (TRAINING SET)
###################################################################
##################

cat("\n========================================\n")
cat("SECTION 12: BENCHMARKING VS OTHER SCORES - TRAINING SET\n")
cat("========================================\n\n")

# Read training data with all scores
# NOTE: This assumes your dataset includes the following score columns:
# CHA2DS2VASc, APPLE, SUCCESS, PAT2C2H, HATCH, BASE_AF2
# If these scores are not in your dataset, they need to be calculated first
```

```r
data_compare_train <- read.csv("AFCA 训练集列线图 2 修改后 ROC nomo vs CHADS.csv")

# Prepare data
data_compare_train$Female <- as.numeric(as.character(data_compare_train$Female))
data_compare_train$PeAF <- as.numeric(as.character(data_compare_train$PeAF))
data_compare_train$PR_interval <-
as.numeric(as.character(data_compare_train$PR_interval))
data_compare_train$LA <- as.numeric(as.character(data_compare_train$LA))
data_compare_train$LAAFV <- as.numeric(as.character(data_compare_train$LAAFV))

# Filter complete cases
complete_cases_compare_train <- complete.cases(
  data_compare_train$follow, data_compare_train$status,
  data_compare_train$Female, data_compare_train$PeAF,
  data_compare_train$PR_interval, data_compare_train$LA,
  data_compare_train$LAAFV
)
model_data_compare_train <- data_compare_train[complete_cases_compare_train, ]

# Build Cox model for nomogram
cox_nomo_train <- coxph(
  Surv(follow, status) ~ Female + PeAF + PR_interval + LA + LAAFV,
  data = model_data_compare_train
)
```

```
# Calculate risk scores
risk_scores_nomo_train <- predict(cox_nomo_train, type = "risk")

# Calculate timeROC for nomogram
roc_nomo_train_12 <- timeROC(
  T = model_data_compare_train$follow,
  delta = model_data_compare_train$status,
  marker = risk_scores_nomo_train,
  cause = 1,
  times = 12,
  iid = TRUE
)

roc_nomo_train_24 <- timeROC(
  T = model_data_compare_train$follow,
  delta = model_data_compare_train$status,
  marker = risk_scores_nomo_train,
  cause = 1,
  times = 24,
  iid = TRUE
)

# Calculate timeROC for other scores
# CHA2DS2-VASc
roc_cha2ds2vasc_train_12 <- timeROC(
  T = model_data_compare_train$follow,
  delta = model_data_compare_train$status,
```

```
  marker = model_data_compare_train$CHA2DS2VASc,
  cause = 1,
  times = 12,
  iid = TRUE
)

roc_cha2ds2vasc_train_24 <- timeROC(
  T = model_data_compare_train$follow,
  delta = model_data_compare_train$status,
  marker = model_data_compare_train$CHA2DS2VASc,
  cause = 1,
  times = 24,
  iid = TRUE
)

cat("\nAUC Results at 12 months (Training Set):\n")
cat("Nomogram:", round(roc_nomo_train_12$AUC[1], 3), "\n")
cat("CHA2DS2-VASc:", round(roc_cha2ds2vasc_train_12$AUC[1], 3), "\n")

cat("\nAUC Results at 24 months (Training Set):\n")
cat("Nomogram:", round(roc_nomo_train_24$AUC[1], 3), "\n")
cat("CHA2DS2-VASc:", round(roc_cha2ds2vasc_train_24$AUC[1], 3), "\n\n")

# NOTE: If you have APPLE, SUCCESS, PAT2C2H, HATCH, BASE-AF2 scores:
# Calculate their timeROC curves similarly

# For demonstration, I'll show the framework for additional scores:
```

```
# Uncomment and modify if you have these scores in your data

# # APPLE Score
# if ("APPLE" %in% colnames(model_data_compare_train)) {
#     roc_apple_train_12 <- timeROC(
#         T = model_data_compare_train$follow,
#         delta = model_data_compare_train$status,
#         marker = model_data_compare_train$APPLE,
#         cause = 1,
#         times = 12,
#         iid = TRUE
#     )
#
#     roc_apple_train_24 <- timeROC(
#         T = model_data_compare_train$follow,
#         delta = model_data_compare_train$status,
#         marker = model_data_compare_train$APPLE,
#         cause = 1,
#         times = 24,
#         iid = TRUE
#     )
#
#     cat("APPLE 12m:", round(roc_apple_train_12$AUC[1], 3), "\n")
#     cat("APPLE 24m:", round(roc_apple_train_24$AUC[1], 3), "\n")
# }

# Plot ROC curves comparison
```

```
par(mar = c(5, 5, 4, 2))
plot(roc_nomo_train_12, time = 12, col = "red", lwd = 4,
     main = "Training Set: 12-month ROC Comparison")
plot(roc_cha2ds2vasc_train_12, time = 12, add = TRUE, col = "blue", lwd = 4)
abline(a = 0, b = 1, lty = 2)

legend("bottomright",
       legend = c(
         paste("Nomogram (AUC =", round(roc_nomo_train_12$AUC[1], 2), ")"),
         paste("CHA2DS2-VASc (AUC =", round(roc_cha2ds2vasc_train_12$AUC[1], 2),
")")
       ),
       col = c("red", "blue"),
       lty = 1,
       lwd = 4,
       bty = "n")

# 24-month comparison
par(mar = c(5, 5, 4, 2))
plot(roc_nomo_train_24, time = 24, col = "red", lwd = 4,
     main = "Training Set: 24-month ROC Comparison")
plot(roc_cha2ds2vasc_train_24, time = 24, add = TRUE, col = "blue", lwd = 4)
abline(a = 0, b = 1, lty = 2)

legend("bottomright",
       legend = c(
         paste("Nomogram (AUC =", round(roc_nomo_train_24$AUC[1], 2), ")"),
```

```
                paste("CHA2DS2-VASc (AUC =", round(roc_cha2ds2vasc_train_24$AUC[1], 2),
")")
            ),
            col = c("red", "blue"),
            lty = 1,
            lwd = 4,
            bty = "n")

###############################################################
#################
# SECTION 13: DELONG TESTS WITH HOLM CORRECTION (TRAINING SET)
###############################################################
#################

cat("\n========================================\n")
cat("SECTION 13: DELONG TESTS - TRAINING SET\n")
cat("========================================\n\n")

cat("Performing paired DeLong tests with Holm adjustment...\n\n")

# Note: For DeLong tests, we need to use pROC package
# We'll need to convert timeROC results to pROC format

# Function to perform DeLong test for time-dependent ROC
perform_delong_test <- function(data, time_point, marker1, marker2,
                                marker1_name, marker2_name) {
  # For time-dependent ROC, we need to calculate case-control status at timepoint
  # This is a simplified approach - for production use, consider more sophisticated
```

methods

```
  # Create time-dependent case-control status
  cases <- data$status == 1 & data$follow <= time_point
  controls <- data$follow > time_point

  # Create subset with valid observations
  valid_obs <- cases | controls

  if (sum(cases) < 10 || sum(controls) < 10) {
    cat("Warning: Insufficient cases or controls for", marker1_name, "vs",
        marker2_name, "at", time_point, "months\n")
    return(NA)
  }

  tryCatch({
    roc1 <- roc(cases[valid_obs], marker1[valid_obs], quiet = TRUE)
    roc2 <- roc(cases[valid_obs], marker2[valid_obs], quiet = TRUE)

    test_result <- roc.test(roc1, roc2, method = "delong", paired = TRUE)

    return(test_result$p.value)
  }, error = function(e) {
    cat("Error in DeLong test:", e$message, "\n")
    return(NA)
  })
}
```

```
# Perform DeLong tests at 12 months
cat("12-month comparisons:\n")

# Nomogram vs CHA2DS2-VASc
p_12_cha2ds2vasc_train <- perform_delong_test(
  data = model_data_compare_train,
  time_point = 12,
  marker1 = risk_scores_nomo_train,
  marker2 = model_data_compare_train$CHA2DS2VASc,
  marker1_name = "Nomogram",
  marker2_name = "CHA2DS2-VASc"
)

cat("Nomogram vs CHA2DS2-VASc: p =", format.pval(p_12_cha2ds2vasc_train, digits = 3), "\n")

# Collect all p-values for Holm adjustment
p_values_12_train <- c(p_12_cha2ds2vasc_train)
comparison_names_12_train <- c("CHA2DS2-VASc")

# Add more comparisons if other scores are available
# Uncomment and modify as needed:
# if ("APPLE" %in% colnames(model_data_compare_train)) {
#     p_12_apple_train <- perform_delong_test(...)
#     p_values_12_train <- c(p_values_12_train, p_12_apple_train)
#     comparison_names_12_train <- c(comparison_names_12_train, "APPLE")
```

```
# }

# Apply Holm correction
p_adjusted_12_train <- p.adjust(p_values_12_train, method = "holm")

cat("\nHolm-adjusted p-values (12 months):\n")
for (i in 1:length(comparison_names_12_train)) {
  cat(comparison_names_12_train[i], ": p.adj =",
        format.pval(p_adjusted_12_train[i], digits = 3), "\n")
}

# Perform DeLong tests at 24 months
cat("\n24-month comparisons:\n")

p_24_cha2ds2vasc_train <- perform_delong_test(
  data = model_data_compare_train,
  time_point = 24,
  marker1 = risk_scores_nomo_train,
  marker2 = model_data_compare_train$CHA2DS2VASc,
  marker1_name = "Nomogram",
  marker2_name = "CHA2DS2-VASc"
)

cat("Nomogram vs CHA2DS2-VASc: p =", format.pval(p_24_cha2ds2vasc_train, digits = 
3), "\n")

p_values_24_train <- c(p_24_cha2ds2vasc_train)
```

```
comparison_names_24_train <- c("CHA2DS2-VASc")

# Apply Holm correction
p_adjusted_24_train <- p.adjust(p_values_24_train, method = "holm")

cat("\nHolm-adjusted p-values (24 months):\n")
for (i in 1:length(comparison_names_24_train)) {
  cat(comparison_names_24_train[i], ": p.adj =",
      format.pval(p_adjusted_24_train[i], digits = 3), "\n")
}

# Create summary table
cat("\n=== SUMMARY TABLE (Training Set) ===\n")
summary_train <- data.frame(
  Comparison = c(paste("Nomogram vs", comparison_names_12_train)),
  AUC_12m_Nomo = round(roc_nomo_train_12$AUC[1], 3),
  AUC_12m_Comparator = round(c(roc_cha2ds2vasc_train_12$AUC[1]), 3),
  P_12m_raw = format.pval(p_values_12_train, digits = 3),
  P_12m_Holm = format.pval(p_adjusted_12_train, digits = 3),
  AUC_24m_Nomo = round(roc_nomo_train_24$AUC[1], 3),
  AUC_24m_Comparator = round(c(roc_cha2ds2vasc_train_24$AUC[1]), 3),
  P_24m_raw = format.pval(p_values_24_train, digits = 3),
  P_24m_Holm = format.pval(p_adjusted_24_train, digits = 3)
)

print(summary_train)
```

```r
# Save summary table

write.csv(summary_train, "Training_Set_DeLong_Results.csv", row.names = FALSE)


###############################################################
#################

# SECTION 14: COMPARISON WITH OTHER SCORES (VALIDATION SET)

###############################################################
#################


cat("\n========================================\n")

cat("SECTION 14: BENCHMARKING VS OTHER SCORES - VALIDATION SET\n")

cat("========================================\n\n")


# Read validation data

data_compare_val <- read.csv("AFCA 验证集列线图修改后.csv")


# Prepare data

data_compare_val$Female <- as.numeric(as.character(data_compare_val$Female))

data_compare_val$PeAF <- as.numeric(as.character(data_compare_val$PeAF))

data_compare_val$PR_interval <-
as.numeric(as.character(data_compare_val$PR_interval))

data_compare_val$LA <- as.numeric(as.character(data_compare_val$LA))

data_compare_val$LAAFV <- as.numeric(as.character(data_compare_val$LAAFV))


# Filter complete cases

complete_cases_compare_val <- complete.cases(

  data_compare_val$follow, data_compare_val$status,

  data_compare_val$Female, data_compare_val$PeAF,
```

```
    data_compare_val$PR_interval, data_compare_val$LA,
    data_compare_val$LAAFV
)
model_data_compare_val <- data_compare_val[complete_cases_compare_val, ]

# Build Cox model
cox_nomo_val <- coxph(
    Surv(follow, status) ~ Female + PeAF + PR_interval + LA + LAAFV,
    data = model_data_compare_val
)

# Calculate risk scores
risk_scores_nomo_val <- predict(cox_nomo_val, type = "risk")

# Calculate timeROC for nomogram
roc_nomo_val_12 <- timeROC(
    T = model_data_compare_val$follow,
    delta = model_data_compare_val$status,
    marker = risk_scores_nomo_val,
    cause = 1,
    times = 12,
    iid = TRUE
)

roc_nomo_val_24 <- timeROC(
    T = model_data_compare_val$follow,
    delta = model_data_compare_val$status,
```

```
  marker = risk_scores_nomo_val,
  cause = 1,
  times = 24,
  iid = TRUE
)

# Calculate timeROC for CHA2DS2-VASc
roc_cha2ds2vasc_val_12 <- timeROC(
  T = model_data_compare_val$follow,
  delta = model_data_compare_val$status,
  marker = model_data_compare_val$CHA2DS2VASc,
  cause = 1,
  times = 12,
  iid = TRUE
)

roc_cha2ds2vasc_val_24 <- timeROC(
  T = model_data_compare_val$follow,
  delta = model_data_compare_val$status,
  marker = model_data_compare_val$CHA2DS2VASc,
  cause = 1,
  times = 24,
  iid = TRUE
)

cat("\nAUC Results at 12 months (Validation Set):\n")
cat("Nomogram:", round(roc_nomo_val_12$AUC[1], 3), "\n")
```

```
cat("CHA2DS2-VASc:", round(roc_cha2ds2vasc_val_12$AUC[1], 3), "\n")

cat("\nAUC Results at 24 months (Validation Set):\n")
cat("Nomogram:", round(roc_nomo_val_24$AUC[1], 3), "\n")
cat("CHA2DS2-VASc:", round(roc_cha2ds2vasc_val_24$AUC[1], 3), "\n\n")

# Plot ROC curves
par(mar = c(5, 5, 4, 2))
plot(roc_nomo_val_12, time = 12, col = "red", lwd = 4,
     main = "Validation Set: 12-month ROC Comparison")
plot(roc_cha2ds2vasc_val_12, time = 12, add = TRUE, col = "blue", lwd = 4)
abline(a = 0, b = 1, lty = 2)

legend("bottomright",
       legend = c(
         paste("Nomogram (AUC =", round(roc_nomo_val_12$AUC[1], 2), ")"),
         paste("CHA2DS2-VASc (AUC =", round(roc_cha2ds2vasc_val_12$AUC[1], 2),
")")
       ),
       col = c("red", "blue"),
       lty = 1,
       lwd = 4,
       bty = "n")

# 24-month comparison
par(mar = c(5, 5, 4, 2))
plot(roc_nomo_val_24, time = 24, col = "red", lwd = 4,
```

```
        main = "Validation Set: 24-month ROC Comparison")
plot(roc_cha2ds2vasc_val_24, time = 24, add = TRUE, col = "blue", lwd = 4)
abline(a = 0, b = 1, lty = 2)

legend("bottomright",
           legend = c(
               paste("Nomogram (AUC =", round(roc_nomo_val_24$AUC[1], 2), ")"),
               paste("CHA2DS2-VASc (AUC =", round(roc_cha2ds2vasc_val_24$AUC[1], 2),
")")
           ),
           col = c("red", "blue"),
           lty = 1,
           lwd = 4,
           bty = "n")

#################################################################
#################
# SECTION 15: DELONG TESTS WITH HOLM CORRECTION (VALIDATION SET)
#################################################################
#################

cat("\n=========================================\n")
cat("SECTION 15: DELONG TESTS - VALIDATION SET\n")
cat("=========================================\n\n")

cat("Performing paired DeLong tests with Holm adjustment...\n\n")

# 12-month comparisons
```

```
cat("12-month comparisons:\n")

p_12_cha2ds2vasc_val <- perform_delong_test(
   data = model_data_compare_val,
   time_point = 12,
   marker1 = risk_scores_nomo_val,
   marker2 = model_data_compare_val$CHA2DS2VASc,
   marker1_name = "Nomogram",
   marker2_name = "CHA2DS2-VASc"
)

cat("Nomogram vs CHA2DS2-VASc: p =", format.pval(p_12_cha2ds2vasc_val, digits = 3),
"\n")

p_values_12_val <- c(p_12_cha2ds2vasc_val)
comparison_names_12_val <- c("CHA2DS2-VASc")

# Apply Holm correction
p_adjusted_12_val <- p.adjust(p_values_12_val, method = "holm")

cat("\nHolm-adjusted p-values (12 months):\n")
for (i in 1:length(comparison_names_12_val)) {
   cat(comparison_names_12_val[i], ": p.adj =",
         format.pval(p_adjusted_12_val[i], digits = 3), "\n")
}

# 24-month comparisons
```

```r
cat("\n24-month comparisons:\n")

p_24_cha2ds2vasc_val <- perform_delong_test(
  data = model_data_compare_val,
  time_point = 24,
  marker1 = risk_scores_nomo_val,
  marker2 = model_data_compare_val$CHA2DS2VASc,
  marker1_name = "Nomogram",
  marker2_name = "CHA2DS2-VASc"
)

cat("Nomogram vs CHA2DS2-VASc: p =", format.pval(p_24_cha2ds2vasc_val, digits = 3),
"\n")

p_values_24_val <- c(p_24_cha2ds2vasc_val)
comparison_names_24_val <- c("CHA2DS2-VASc")

# Apply Holm correction
p_adjusted_24_val <- p.adjust(p_values_24_val, method = "holm")

cat("\nHolm-adjusted p-values (24 months):\n")
for (i in 1:length(comparison_names_24_val)) {
  cat(comparison_names_24_val[i], ": p.adj =",
      format.pval(p_adjusted_24_val[i], digits = 3), "\n")
}

# Create summary table
```

```r
cat("\n=== SUMMARY TABLE (Validation Set) ===\n")
summary_val <- data.frame(
  Comparison = c(paste("Nomogram vs", comparison_names_12_val)),
  AUC_12m_Nomo = round(roc_nomo_val_12$AUC[1], 3),
  AUC_12m_Comparator = round(c(roc_cha2ds2vasc_val_12$AUC[1]), 3),
  P_12m_raw = format.pval(p_values_12_val, digits = 3),
  P_12m_Holm = format.pval(p_adjusted_12_val, digits = 3),
  AUC_24m_Nomo = round(roc_nomo_val_24$AUC[1], 3),
  AUC_24m_Comparator = round(c(roc_cha2ds2vasc_val_24$AUC[1], 3)),
  P_24m_raw = format.pval(p_values_24_val, digits = 3),
  P_24m_Holm = format.pval(p_adjusted_24_val, digits = 3)
)

print(summary_val)

# Save summary table
write.csv(summary_val, "Validation_Set_DeLong_Results.csv", row.names = FALSE)

###############################################################
#################
# FINAL SUMMARY
###############################################################
#################

cat("\n========================================\n")
cat("ALL ANALYSES COMPLETED SUCCESSFULLY\n")
cat("========================================\n\n")
```

```
cat("Summary of completed analyses:\n")
cat("1. LASSO regression for variable selection\n")
cat("2. Random forest analysis for variable importance\n")
cat("3. Cox regression and nomogram construction\n")
cat("4. Time-dependent ROC curves (training and validation)\n")
cat("5. Calibration curves (training and validation)\n")
cat("6. Decision curve analysis (training and validation)\n")
cat("7. Kaplan-Meier survival analysis with risk stratification\n")
cat("8. Benchmarking against established AF scores\n")
cat("9. Paired DeLong tests with Holm adjustment\n\n")

cat("Output files generated:\n")
cat("- AFCA 训练集列线图计算后患者总点数.csv\n")
cat("- 训练集 DCA.csv\n")
cat("- 验证集 DCA.csv\n")
cat("- Training_Set_DeLong_Results.csv\n")
cat("- Validation_Set_DeLong_Results.csv\n")
cat("- random_forest_top20_importance.png\n\n")

cat("Session info:\n")
print(sessionInfo())

cat("\n=== END OF SCRIPT ===\n")
```
